# School Relational Cohesion, Gender-inequitable Attitudes, and Bullying Perpetration Across 15 Education Systems: Less Bullying Overall Without a Weaker Attitude-behavior Link

**DOI:** 10.1007/s10964-026-02416-8

**Published:** 2026-09-24

**Authors:** Boxuan Song

**Affiliations:** Department of Applied Social Sciences, The Hong Kong Polytechnic University, SAR Hong Kong, China

**Keywords:** Gender-inequitable Attitudes, Bullying Perpetration, School Relational Cohesion, School Climate, Measurement Invariance

## Abstract

Gender-inequitable attitudes are consistently associated with bullying perpetration, yet whether supportive schools weaken the link between those attitudes and behavior, or only lower the overall level of perpetration, remains unclear. Data came from the older cohort of the Survey on Social and Emotional Skills 2023. The analytic sample comprised 47,962 mid-adolescents (49.3% girls, 49.5% boys; M age = 15.76 years, SD = 0.29) in 1,263 schools across 15 education systems, five of them national samples and ten cities or sub-national regions. Two-level structural equation models with site indicators were estimated. Endorsement of gender-inequitable attitudes was positively associated with perpetration, and a more female-concentrated household division of labor was indirectly associated with perpetration through those attitudes. Schools higher in relational cohesion reported less bullying on average, but relational cohesion was not associated with a weaker within-school attitude-behavior link. A more hostile school gender climate was associated with both more bullying and a modestly stronger link. An exploratory gender difference in this moderation was not reproduced in split-sample models. Alignment analysis flagged 48% of gender-attitude parameters as noninvariant across systems.

**Supplementary Information:**

The online version contains supplementary material available at https://doi.org/10.1007/s10964-026-02416-8.

## Introduction

Bullying is among the most common forms of interpersonal harm at school during adolescence (Perino et al., 2025). About one in three students worldwide report having been bullied by peers in the preceding month (Biswas et al., 2020), and the prevalence of perpetration is comparable (Ariani et al., 2025). Both being bullied and bullying others carry consequences that reach into adult mental health, relationships, and socioeconomic attainment (Arseneault, 2018). Bullying is also gendered. Boys perpetrate more than girls, the gap widens in more structurally unequal societies (Cosma et al., 2022), and bullying in early adolescence prospectively predicts later sexual harassment and dating violence perpetration (Espelage et al., 2015). Adolescents who endorse gender-inequitable attitudes bully more (Gao et al., 2022), and supportive schools have less bullying on average (Wang & Degol, 2016). What remains unknown is whether supportive schools weaken the link between a student’s gender-inequitable beliefs and that student’s own bullying, or whether they lower the overall level of bullying while the link between belief and behavior stays intact. Level and slope are rarely estimated in the same model, and the comparability of the gender attitude scales used across education systems is seldom tested. This study addresses both gaps by estimating level and slope together among mid-adolescents in 15 education systems and by testing whether the gender attitude scale is approximately invariant across those systems.

### Bullying and Gender in Mid-adolescence

How bullying operates changes across adolescence, and mid-adolescence, at about age 15, differs from the early secondary years in ways that bear on the gendering of peer aggression (Salmivalli et al., 2021). Although the overall frequency of perpetration tends to decline after the early secondary years, the bullying that persists into mid-adolescence is increasingly strategic and status-driven, sustained by the heightened importance of popularity within the peer group at this age (Romera et al., 2021). Its forms shift accordingly, away from overt physical aggression and toward verbal, relational, and sexualized conduct, so that gender-based harassment and homophobic name-calling rank among the most common vehicles of peer aggression (Espelage et al., 2015). The policing of gender expression that intensifies at the threshold of adolescence (Blum et al., 2017) remains acute through the mid-adolescent years. Emerging romantic and sexual interest raises the salience of gender conformity, and students who depart from gender norms continue to face elevated victimization (Kiekens et al., 2024). Mid-adolescence is therefore a developmental window in which gendered beliefs and bullying at school are especially likely to be coupled.

### Gender-inequitable Attitudes and Peer Violence

Social cognitive accounts of gender development hold that children acquire beliefs about what males and females are and ought to be, and then regulate their own conduct against those internalized standards (Bussey & Bandura, 1999). Gender schema theory makes a parallel claim about how such beliefs are cognitively organized (Bem, 1981; Martin & Ruble, 2004). These expectations intensify sharply at the threshold of adolescence, when peers, parents, and institutions begin to police gender boundaries with new force across a wide range of cultural settings (Blum et al., 2017). Gender norms in this literature are socially enforced rules whose violation carries sanctions, and they operate well beyond the level of private belief (Cislaghi & Heise, 2020). Where the prevailing standard prescribes male dominance, toughness, and the devaluation of anything coded as feminine, what Connell and Messerschmidt (2005) termed hegemonic masculinity, aggression is not a departure from the gender order but a resource for accomplishing it (Reigeluth & Addis, 2016).

The empirical record is broadly consistent with the expectation that gender-inequitable beliefs foster aggression, although the strength of the association depends on what is being predicted. Gender-inequitable attitudes are among the more reliable predictors of violence that is itself gendered, including adolescent dating violence where injunctive norms condone it (Reyes et al., 2016), sexual harassment, and men’s intimate partner violence in adulthood (Heise et al., 2019; Jewkes et al., 2015). Among adolescents specifically, traditional gender role attitudes have also been linked to general bullying perpetration and to homophobic teasing (Gao et al., 2022). Reviews of gender attitudes in early adolescence document their early emergence and their association with violence and delinquency across settings (Kagesten et al., 2016). The association with general bullying is reliable but modest in size, and the strongest attitude to behavior links are still observed for forms of aggression that carry a gender component. Restrictive gender norms are accordingly treated in global health frameworks as a risk factor for violence and ill health in their own right and not as a proxy for other disadvantages (Cislaghi & Heise, 2020). What remains open is whether the strength of this attitude to behavior association is fixed or is conditioned by the school contexts in which adolescents live.

### Family Origins of Adolescents’ Gender Attitudes

If gendered beliefs precede violence, the question becomes where the beliefs originate, and a long line of theory and evidence points first to the family (Davis & Greenstein, 2009; Endendijk et al., 2018). Households enact the gender system in its most visible and repetitive form, in who cooks, who cleans, who looks after children, and who cares for sick relatives. Household labor has been described as the routine production of gender, a daily demonstration through which gender beliefs are enacted as well as reflected (Coltrane, 1989; West & Zimmerman, 1987). Children appear to read this demonstration, and what parents do is at least as strongly associated with children’s gender attitudes as what parents say they believe (Croft et al., 2014; Platt & Polavieja, 2016). Longitudinal and cross-national work converges on the same point. Parents’ enacted division of housework predicts children’s own gender ideology in adulthood (Cunningham, 2001). Children’s contributions to housework reproduce the gendered patterns they observe at home (Cordero-Coma & Esping-Andersen, 2018). Parental gender attitudes and practices transmit to the next generation in settings as different as Britain and India (Dhar et al., 2019; Platt & Polavieja, 2016). These arrangements are themselves embedded in wider institutional configurations of care and paid work (Ji et al., 2017).

Recent evidence connects the family and bullying literatures directly. In an analysis of the Survey on Social and Emotional Skills 2023, adolescents in households with gender-specialized or gender-traditional patterns of labor division reported stronger gender bias (Zheng et al., 2026). That bias was associated with more bullying perpetration, and the association was stronger among boys. That analysis treated adolescents as independent observations. It remains unexamined whether the same sequence operates contextually. Schools whose students come on average from more traditionally organized households may show higher average stereotype endorsement and, through it, higher average perpetration. This is the level at which school-based prevention operates (Bronfenbrenner, 1979).

### Schools as Contexts of Informal Social Control

Neighborhood collective efficacy has been defined as social cohesion combined with a shared willingness to intervene for the common good (Sampson et al., 1997). The construct has two theoretically distinct components, which are cohesion and informal social control, the second being the willingness of members to sanction disorder. Schools are a more tractable case than neighborhoods, because membership is involuntary and stable and because the relational component can be measured directly through students’ reports (Niu, 2025). A supportive relational climate refers to the shared quality of students’ relationships within a school, and is indicated at the school level by the average student report of belonging at school, of relationships with classmates, and of relationships with teachers. Studies applying the broader construct to schools have found that stronger school collective efficacy is associated with less bullying victimization and perpetration (Olsson et al., 2017; Sapouna, 2010). Reviews of school climate report a consistent, if modest, negative association with school violence (Wang & Degol, 2016), and meta-analytic evidence from whole-school prevention shows that changing the school environment reduces perpetration (Gaffney et al. 2021b). This is coherent with the view of bullying as a group process that is sustained or inhibited by the surrounding peer collective (Salmivalli, 2010) and nested within family, school, and community systems (Hong & Espelage, 2012).

Work on authoritative school discipline draws on the parenting styles analogy to characterize schools that combine high structure with high support. Schools rated high on both dimensions report lower rates of student bullying and victimization (Gregory et al., 2010). The pattern extends to student-reported risk behavior more broadly (Cornell & Huang, 2016) and to peer aggression across racial and ethnic groups (Konold et al., 2017). This tradition converges with the collective efficacy literature on the level prediction, in that supportive and structured schools have less peer aggression on average. Whether such schools also weaken the within-school link between individual gender-inequitable beliefs and individual bullying has not been examined systematically.

Supportive schools could also have less bullying without any change in how individual beliefs translate into behavior. Relational cohesion may be associated with the general level of aggression through supervision, opportunity structure, and normative climate, while showing no association with how tightly beliefs and behavior are coupled within individuals. Classroom-level norms have been found to account for variance in bullying-related behavior at the classroom level, while individual attitudes continued to predict behavior at the student level (Salmivalli & Voeten, 2004). Later work on classroom peer ecologies has located contextual effects mainly at the level of group rates and not at the level of individual couplings (Saarento & Salmivalli, 2015).

### Gender, Composition, and Climate as Boundary Conditions

The same relational environment need not operate identically for boys and girls. The class context has been reported to bear more strongly on girls’ than on boys’ bullying-related behavior (Salmivalli & Voeten, 2004). Girls’ aggression is more often relational in form and therefore more deeply embedded in the very ties that a cohesive school strengthens and renders visible (Card et al., 2008; Rose & Rudolph, 2006), so relational monitoring is more likely to reach it. For boys, a cohesive and densely connected setting may be where masculinity is most publicly adjudicated. Dense peer ties can then intensify status competition, and a student who already endorses a dominance-based view of gender finds more occasions on which to enact it (Reigeluth & Addis, 2016). These mechanisms imply cross-level moderation of opposite sign for the two groups, which a pooled estimate could conceal.

A school-level association between relational climate and bullying can look relational without being so. Schools differ in socioeconomic profile, gender composition, and immigrant density, and a measure built from students’ relational reports may partly index who attends the school and not how the school works (Wang & Degol, 2016). A distinct rival account locates the operative contextual risk in the presence of gender hostility. Perceived gender-based harassment and disrespect is conceptually separable from low relational quality (Rinehart & Espelage, 2016). Recent cross-national evidence indicates that school-based gender-based violence is positively associated with bullying perpetration, partly through reductions in adolescents’ social and emotional competence, and more strongly so in higher-masculinity national cultures (Jiang et al., 2026).

### Measuring Gender Attitudes Across Education Systems

Comparing gender attitudes across education systems also raises a measurement question. Cross-national research on gender attitudes routinely assumes that a scale means the same thing in every site, yet this assumption is seldom tested (Putnick & Bornstein, 2016). Scale-development work in early adolescence has shown how difficult it is to build gender norm measures that travel across cultural settings (Kagesten et al., 2016; Moreau et al., 2019). Conventional multiple group confirmatory factor analysis becomes impractical when the number of groups is large. The alignment method makes approximate invariance testing feasible with many groups by allowing latent means and variances to vary while minimizing total noninvariance, against a conventional benchmark of no more than 25% noninvariant parameters (Asparouhov & Muthen, 2014; Luong & Flake, 2023). Whether a widely fielded gender attitude scale meets this benchmark bears on every cross-system comparison. If the scale is not approximately invariant, comparisons of latent means across systems are not interpretable, whatever the analysis of associations shows.

## Current Study

Level and slope are rarely estimated in the same model, so it is not known whether supportive schools weaken the link between gender-inequitable beliefs and bullying or only lower its level. This study estimates both in the same models and tests whether the gender attitude scale is approximately invariant across the 15 education systems. Endorsement of gender-inequitable attitudes was expected to be positively associated with bullying perpetration (Hypothesis 1). Because households model gender arrangements, a more traditional division of household labor was expected to be indirectly associated with perpetration through those attitudes at the student and school levels (Hypothesis 2). School relational cohesion was expected to be associated with lower average perpetration (Hypothesis 3). Under a buffering account it was also expected to weaken the within-school slope of perpetration on attitudes (Hypothesis 4), although an account in which context operates on rates alone predicts no such moderation. Because relational monitoring and status competition may run in opposite directions for girls and boys, that moderation was expected to differ by gender (Hypothesis 5). A more hostile school gender climate was expected to be associated with more perpetration independently of relational cohesion (Hypothesis 6).

## Methods

### Participants and Procedure

Data were drawn from the second round of the Organisation for Economic Co-operation and Development (OECD) Survey on Social and Emotional Skills (SSES) 2023, an international study of adolescents’ social and emotional skills and the family, school, and community contexts in which those skills develop (OECD, 2024, 2025). The present analyses used the older cohort, comprising students in mid-adolescence, because this cohort was assessed at every participating site and provided the school gender-violence-climate index that was central to the contextual models. The older cohort was administered in 16 education systems. Five were national samples, namely Bulgaria, Chile, Peru, Spain, and Ukraine, the last covering 19 of that country’s 27 regions. Ten were sub-national entities, namely Bogotá (Colombia), Delhi (India), Dubai (United Arab Emirates), Emilia-Romagna (Italy), Gunma (Japan), Helsinki (Finland), Jinan (China), Kudus (Indonesia), Sobral (Brazil), and Turin (Italy). The sixteenth system, Mexico, is not analyzed here, because the OECD reports Mexican data separately from the international average after the sampling frame proved inconsistent with independent estimates of the eligible population and after school participation, teacher response, and several administrative standards fell below the technical requirements (OECD, 2025). The analytic sample therefore covers 15 systems that are a mixture of countries, regions, and cities, and the phrase across 15 education systems should not be read as across 15 countries. These 15 systems are referred to below as sites. The analytic sample comprised 47,962 students nested within 1,263 schools, with a mean age of 15.76 years (SD = 0.29, range 15.18 to 16.34). Of these students, 23,763 were boys and 23,647 were girls, and 552 students who reported another category or declined to answer were set to missing on gender. Sampled students per school averaged 38.0 (SD = 20.3). Students completed a self-administered questionnaire that included the social and emotional skill scales together with attitudinal, relational, and background items, and school principals completed a separate questionnaire that provided school-level information on safety and anti-bullying provision. Because the study used de-identified secondary data released for research use, no additional ethical approval was required. Sampling weights supplied with the data were applied in sensitivity analyses reported below. The number of students and schools contributing to each system is reported in Appendix Table A6.

### Measures

Each multi-item construct was represented by an index that the OECD constructed with an item response theory model of the generalized partial credit type, expressed as a weighted likelihood estimate, adjusted for acquiescence, and standardized to the SSES international reporting metric using the older cohort as the reference group. Table 1 reports each index on the OECD international reporting metric, so that descriptive values remain interpretable against the reference population. For the substantive models, all continuous variables were standardized within the analytic sample to a mean of 0 and a standard deviation of 1, so that regression coefficients are interpretable in standard-deviation units.

**Table 1:** Descriptive Statistics

Panel A. Student level			
Variable	Boys M (SD) / %	Girls M (SD) / %	SMD
Perpetration	0.18 (1.08)	-0.19 (0.87)	0.38
Gender-bias attitudes	0.26 (0.96)	-0.25 (0.97)	0.53
Agentic/status subdimension	0.39 (0.95)	-0.38 (0.89)	0.84
Communal/care subdimension	0.12 (0.91)	-0.11 (1.06)	0.23
Home female roles	-0.03 (0.97)	0.03 (1.03)	-0.06
Socioeconomic status	0.04 (0.99)	-0.04 (1.01)	0.08
Acquiescence	-0.04 (1.04)	0.05 (0.94)	-0.09
Age	15.76 (0.29)	15.76 (0.29)	0.01
Immigrant background (%)	16.9	16.3	0.02
Non-test home language (%)	27.5	27.8	-0.01
**Panel B. School level**			
**Variable**	**M (SD)**	**Min-Max**	**n schools**
School relational cohesion	0.00 (1.00)	-3.61 to 5.32	1,262
School institutional order	0.00 (1.00)	-3.38 to 1.62	1,172
School mean SES	0.00 (1.00)	-2.53 to 1.99	1,263
School % girls	0.00 (1.00)	-2.31 to 2.36	1,263
School % immigrant	0.00 (1.00)	-0.59 to 2.96	1,260
School gender-violence climate	0.00 (1.00)	-5.06 to 2.50	1,262
Sampled students per school	38.0 (20.3)	1-360	1,263

Note. Student-level indices are reported on the OECD international reporting metric, on which each index has a mean of 0 and a standard deviation of 1 in the pooled older-cohort reference population; they are not additionally standardized within the analytic sample. Age is reported in raw years. Student-level ns range 21,966–23,763 (boys) and 22,036–23,647 (girls) across variables; 552 students are missing gender. SMD = (M_boys − M_girls) / pooled SD; positive values indicate higher scores for boys. School-level variables are standardized across schools (one record per school); school institutional order is available for 1,172 of 1,263 schools because it draws on principal reports

#### Bullying Perpetration

The outcome was the bullying perpetration index (ST_BULLYPERP), derived from six items (question STQM158) that asked how often, during the school year, the student had engaged in specific aggressive behaviors toward other students. Items covered relational, verbal, and physical forms, for example “I threatened another student” and “I hit or pushed other students around,” each rated on a four-point frequency scale ranging from never or almost never to once a week or more. Higher scores indicate more frequent perpetration. Because responses are anchored on the frequency of behavior across the school year, reports in the two higher categories capture the repeated character that classic definitions of bullying require (Olweus, 1993). The scale does not contain a direct measure of the power imbalance criterion, which is taken up in the Limitations. The OECD documentation reports metric invariance for this scale across sites, which supports the comparison of regression slopes across systems. Response distributions for the six items, overall and by gender, are reported in Appendix Table A5

#### Gender-bias Attitudes 

The mediator was an eight-item index of attitudes toward gender stereotypes (ST_GENBIAS, question STQM146), for example “Boys are more ambitious than girls” and “Women are better prepared to care for children than men,” each rated on a five-point agreement scale. No items were reverse worded, and higher scores indicate stronger endorsement of gender stereotypes. The scale is referred to as gender-bias attitudes in the Methods, Results, tables, and appendix, following the name of the OECD index, and as gender-inequitable attitudes where the construct rather than the instrument is at issue; the two terms denote the same measure. Following the stereotype content literature (Fiske et al., 2002), the eight items fall into two subdimensions. Four items concern agentic and status attributes, covering ambition, leadership, technical ability, and aggressiveness, and four concern communal and care attributes, covering caregiving, empathy, artistic interests, and sensitivity. Because a single factor solution fit these items poorly, this two-dimensional structure was examined empirically before the substantive models were estimated, and the results appear in Table 2

**Table 2:** Measurement of Gender-Bias Attitudes: CFA and Cross-Site Alignment

Panel A. Two-factor CFA of gender-bias items (with ARS control)			
	Agentic and status (λ)	Communal and care (λ)	
Ambition	0.655	—	
Leadership	0.852	—	
Technology	0.857	—	
Aggressiveness	0.510	—	
Caring	—	0.729	
Empathy	—	0.751	
Arts	—	0.754	
Sensitivity	—	0.741	
McDonald’s ω	0.817	0.832	
ARS → factor (STDYX)	0.135***	0.206***	
Factor correlation (STDYX)	0.654***		
Panel B. Alignment (free) across 15 sites, one factor			
	Noninvariant	Total	%
Item intercepts	61	120	50.8
Factor loadings	55	120	45.8
All measurement parameters	116	240	48.3

Model fit: χ²(25) = 5126.38 (MLR), CFI = .943, TLI = .917, RMSEA = .066 [90% CI .064, .067], SRMR = .059, N = 47,165

*Note*. Panel A: MLR with cluster-robust SEs (TYPE = COMPLEX; 1,263 schools); standardized (STDYX) loadings; both factors regressed on the acquiescence index (ARS). ω computed from standardized loadings and residual variances. Panel B: free alignment, MLR, 15 known classes; parenthesized entries in the Mplus invariance table were counted as noninvariant. The share of noninvariant parameters (48.3%) far exceeds the 25% benchmark (Muthen & Asparouhov, 2014), so latent means were not compared across sites and attitudinal scores were standardized within the analytic sample; note that significance-based flags are highly sensitive at N= 47,165. Mexico, the sixteenth education system in which the older cohort was administered, is not analyzed, because the OECD reports Mexican data separately from the international average after sampling and technical standards were not met (OECD, 2025). ***p< .001

#### Home Female Roles

The family predictor was the home female-roles index (ST_HOMGEN), based on four items (question STQM149) that asked which family members usually perform preparing meals, looking after children, cleaning the house, and caring for sick family members. Higher scores indicate a more traditional, female-concentrated division of domestic labor. The OECD documentation reports metric invariance for this scale across sites

#### School Relational Cohesion

The focal school-level moderator represented the shared, supportive relational environment of the school and corresponds to the cohesion component of collective efficacy. It was formed from the school means of three student-reported relational scales, all of which reached metric invariance across sites, namely sense of belonging at school (ST_BELONG), relationships with classmates (ST_STUCLASS), and perceived relationships with teachers (ST_RELTEACH). Each student scale was standardized, aggregated to the school mean, standardized across schools, and then averaged, and the resulting composite was standardized across schools. Higher scores indicate a more cohesive and supportive relational climate. The willingness of students and staff to intervene against aggression, which is the second component of collective efficacy in its original formulation, is not measured in SSES 2023. The composite is therefore named and interpreted as relational cohesion throughout, and the interpretive limits that this imposes are set out in the Discussion

#### Additional School-level and Individual Covariates

Three further school-level variables were included, each standardized across schools. School gender-violence climate was the school mean of the gender-based-violence index (ST_GENVIO, question STQM150; available for the older cohort only), whose items concern whether students at the school are treated with respect regardless of gender and the frequency of gender-based ridicule and sexually inappropriate conduct, with higher scores indicating a more hostile climate. School institutional order averaged principal-reported school safety (PR_SCHSAFT, reverse scored so that higher values indicate a safer and more orderly setting) and the count of reported anti-bullying measures (PR_REPBULLY). This composite indexes formal social control and therefore complements the relational cohesion measure, which indexes cohesion rather than the informal enforcement of norms. It is treated as secondary because principal reports were available for a subset of schools and were not compared across sites. School composition was captured by school mean socioeconomic status (SES), proportion female, and immigrant density, each aggregated from student reports. At the individual level, models adjusted for student gender, which the survey records as a student’s own report of their gender rather than as an administrative or biological classification, coded 1 for girls and 0 for boys, with students reporting another category or declining to answer set to missing. Models also adjusted for SES (OECD composite index), immigrant background, speaking a language other than the language of assessment at home, age, and the OECD acquiescence score, the last included so that associations involving attitudinal reports are adjusted for a general tendency to agree

### Statistical Analysis

All models were estimated on cross-sectional data. Indirect coefficients in the mediation and moderated mediation models should therefore be read as decompositions of within-school and between-school covariance and not as evidence of a temporal sequence. Reverse and reciprocal pathways among household arrangements, attitudes, and behavior are equally consistent with the observed covariances. This qualification is stated here, before any indirect coefficient is reported, and it applies to every mediation result below. The substantive contribution of the analysis lies primarily in the moderation models, whose interpretation does not depend on temporal ordering.

Analyses used two-level multilevel structural equation models, with students at Level 1 nested within schools at Level 2, estimated in Mplus 8.3. Models were estimated with maximum likelihood and robust standard errors, and missing data on endogenous variables were handled with full-information maximum likelihood. The Bayesian estimator was used for the moderated mediation model, because that specification places a random slope on an endogenous mediator with a latent decomposition of within- and between-school variance, which the maximum-likelihood estimator does not accommodate. Markov chain Monte Carlo settings and convergence diagnostics are reported in the Appendix. Site indicators, comprising 14 dummy variables for the 15 participating education systems with one system as the reference, were entered at the school level as fixed effects, so that all coefficients describe associations within an education system and stable between system differences in prevalence and reporting are absorbed. The term site fixed effects is used throughout in this sense. Because the data are cross-sectional, no time-varying interpretation is intended. Individual predictors were entered at the within level and group-mean centered, with their school means and the purely school-level variables entered at the between level and treated, where the estimator permitted, as latent aggregates (Enders & Tofighi, 2007; Preacher et al., 2010).

Preliminary analyses evaluated the multilevel structure and the measurement of the gender-bias scale. The intraclass correlation of bullying perpetration was estimated from an unconditional two-level model. The gender-bias items were examined with confirmatory factor analysis, comparing a single-factor solution with a two-factor solution that distinguished agentic and status stereotypes (items STQM14601 to STQM14604) from communal and care stereotypes (items STQM14605 to STQM14608), each with the acquiescence score entered as a covariate. Approximate cross-site invariance of the gender-bias items was then examined with the alignment method across the 15 older-cohort sites, and the proportion of noninvariant intercept and loading parameters was compared with the conventional 25% benchmark. Internal consistency was quantified with McDonald’s omega estimated from the congeneric two-factor model, with its tau-equivalent counterpart computed for comparison (McNeish, 2018), both in the pooled sample and separately within each of the 15 sites (Appendix Table A1). The reliability of aggregating the three relational scales to the school level was evaluated with the intraclass correlation coefficients ICC(1) and ICC(2) from unconditional two-level models (Bliese, 2000; Geldhof et al., 2014; Appendix Table A2).

The substantive models were estimated in sequence and are reported in Table 3. Model 1 is a random intercept baseline relating gender-bias attitudes to perpetration with individual covariates and site fixed effects. Model 2 is a 1-1-1 mediation model decomposing the indirect association of home female roles with perpetration through gender-bias attitudes into within-school and between-school components. Model 3 is a random slope model regressing the attitude to perpetration slope on school relational cohesion and on the secondary school-level moderators. Model 4 adds a within-level product of female and attitudes together with its cross-level moderation. Model 5 adds school gender violence climate, both as a predictor and as a moderator of the slope, among older-cohort schools. Model 6 is a Bayesian moderated mediation model yielding conditional indirect associations and an index of moderated mediation (Table 4). Each interaction was probed at one standard deviation below and above the mean of the moderator (Appendix Table A3).

**Table 3:** Two-Level Models Predicting Bullying Perpetration (M1–M5)

Parameter	M1	M2	M3	M4	M5
***Fixed effects: within level (individual)***					
Gender-bias attitudes → perpetration (mean slope)	0.078 (0.006)***	0.068 (0.006)***	0.074 (0.006)***	0.107 (0.009)***	0.075 (0.006)***
× Female (within slope difference)	—	—	—	− 0.056 (0.011)***	—
Home female roles → perpetration (direct)	—	0.050 (0.005)***	—	—	—
Home female roles → attitudes (a path)	—	0.118 (0.006)***	—	—	—
Female	− 0.324 (0.010)***	− 0.318 (0.010)***	− 0.311 (0.011)***	− 0.312 (0.011)***	− 0.310 (0.011)***
Socio-economic status	0.041 (0.007)***	0.044 (0.007)***	0.042 (0.007)***	0.042 (0.008)***	0.042 (0.007)***
Immigrant background	0.042 (0.021)*	0.045 (0.021)*	0.049 (0.022)*	0.050 (0.022)*	0.049 (0.022)*
Home language other than test language	0.095 (0.014)***	0.090 (0.014)***	0.094 (0.015)***	0.093 (0.015)***	0.096 (0.015)***
Age	− 0.004 (0.005)	− 0.004 (0.005)	− 0.003 (0.005)	− 0.003 (0.005)	− 0.002 (0.005)
Acquiescence response style	0.037 (0.007)***	0.031 (0.007)***	0.039 (0.007)***	0.038 (0.007)***	0.038 (0.007)***
***Fixed effects: between level (school)***					
Gender-bias attitudes (school) → perpetration	0.287 (0.051)***	0.216 (0.037)***	0.153 (0.050)**	0.180 (0.101)†	0.071 (0.055)
Home female roles (school) → perpetration	—	0.119 (0.097)	—	—	—
School relational cohesion → perpetration	—	—	− 0.101 (0.010)***	− 0.107 (0.010)***	− 0.066 (0.012)***
School institutional order → perpetration	—	—	0.008 (0.008)	0.008 (0.008)	0.009 (0.008)
School mean SES → perpetration	—	—	0.028 (0.014)*	0.020 (0.014)	0.027 (0.014)*
School proportion female → perpetration	—	—	− 0.020 (0.008)**	− 0.013 (0.008)	− 0.007 (0.008)
School immigrant density → perpetration	—	—	− 0.003 (0.016)	− 0.006 (0.016)	− 0.001 (0.016)
School gender-violence climate → perpetration	—	—	—	—	0.079 (0.015)***
Site fixed effects	Yes	Yes	Yes	Yes	Yes
***Cross-level interaction (predicting attitudes → perpetration slope)***					
School relational cohesion → slope	—	—	0.004 (0.007)	0.018 (0.010)†	0.013 (0.009)
× Female (three-way)	—	—	—	− 0.025 (0.012)*	—
School institutional order → slope	—	—	0.000 (0.006)	—	0.002 (0.006)
School mean SES → slope	—	—	0.006 (0.007)	—	0.002 (0.007)
School gender-violence climate → slope	—	—	—	—	0.016 (0.007)*
***Variance components***					
Residual variance (within)	0.809	0.787	0.791	0.785	0.791
Intercept variance (between)	0.027	0.025	0.017	0.019	0.016
Slope variance (attitudes)	—	—	0.007	0.023	0.007
Female-slope variance	—	—	—	0.019	—
***Model information***					
Estimator	MLR	MLR	MLR	MLR	MLR
Observations	40,880	41,145	38,198	38,198	38,198
Schools	1,253	1,254	1,163	1,163	1,163
Log-likelihood	-107,346	-164,271	-99,939	-87,590	-99,868

Note. Unstandardized coefficients with robust standard errors in parentheses; all variables were standardized within the analytic sample (M = 0, SD = 1), so coefficients are in standard-deviation units. Estimator is MLR with full-information maximum likelihood for missing data on endogenous variables. All models include site fixed effects (site 1 = reference); site coefficients are omitted. Analytic Ns vary with missing data on individual-level covariates, from 38,198 students in 1,163 schools (Model 5) to 41,145 students in 1,254 schools (Model 2). Model 2 adds the mediation pathway (indirect effects in Appendix Table A3); Models 3–5 add a random slope for the attitudes–perpetration association; Model 4 adds Female × attitudes and its cross-level moderation; Model 5 (older-cohort schools) adds school gender-violence climate. Dashes indicate terms not included in that model. * *p* < .05. ** *p* < .01. *** *p* < .001

**Table 4:** Multilevel Moderated Mediation: Home Female Roles → Gender-Bias Attitudes → Bullying Perpetration, With the Attitudes-Perpetration Slope Moderated by School Relational Cohesion (Model 6)

Parameter	Estimate	Posterior SD	95% credible interval
***Structural paths: within level (individual)***			
Home female roles → gender-bias attitudes (a path)	0.122*	0.005	[0.112, 0.132]
Gender-bias attitudes → perpetration (mean random slope, b path)	0.068*	0.006	[0.055, 0.080]
Home female roles → perpetration (direct)	0.053*	0.005	[0.043, 0.063]
Female	− 0.317*	0.010	[-0.337, − 0.297]
Socio-economic status	0.046*	0.007	[0.033, 0.059]
Immigrant background	0.050*	0.020	[0.012, 0.089]
Home language other than test language	0.092*	0.014	[0.065, 0.119]
Age	− 0.004	0.005	[-0.014, 0.005]
Acquiescence response style	0.033*	0.005	[0.022, 0.043]
***Structural paths: between level (school)***			
Home female roles → gender-bias attitudes (a path)	0.698*	0.039	[0.622, 0.774]
Gender-bias attitudes → perpetration (b path)	0.155*	0.038	[0.078, 0.228]
Home female roles → perpetration (direct)	0.056	0.047	[-0.038, 0.149]
School relational cohesion → perpetration	− 0.104*	0.009	[-0.122, − 0.086]
School institutional order → perpetration	0.010	0.008	[-0.006, 0.026]
School mean SES → perpetration	0.017	0.013	[-0.009, 0.043]
***Moderation of the attitudes-perpetration slope***			
School relational cohesion → slope (b1)	0.010	0.006	[-0.002, 0.023]
School institutional order → slope	− 0.001	0.006	[-0.013, 0.010]
School mean SES → slope	0.006	0.007	[-0.007, 0.020]
***Conditional indirect effects (home roles → attitudes → perpetration)***			
At low relational cohesion (-1 SD)	0.007*	0.001	[0.005, 0.009]
At mean relational cohesion	0.008*	0.001	[0.007, 0.010]
At high relational cohesion (+ 1 SD)	0.009*	0.001	[0.007, 0.012]
Index of moderated mediation (2 × a × b1)	0.002	0.002	[0.000, 0.006]
***Model information***			
Estimator	Bayes (2 chains, 50,000 iterations)		
Deviance information criterion (DIC)	310,969		
Observations / schools	39,146 / 1,166		

Note. Posterior medians from Bayesian estimation (two MCMC chains, 50,000 iterations, potential scale reduction < 1.05); all variables were standardized within the analytic sample, and site fixed effects are included. The a path is home female roles → gender-bias attitudes, the b path is the attitudes → perpetration random slope, and the direct path is home female roles → perpetration. Conditional indirect effects are the product of the a path and the b path implied at each level of school relational cohesion; the index of moderated mediation quantifies the difference in the indirect association between schools one standard deviation above and below the mean on school relational cohesion. Asterisks denote 95% credible intervals excluding zero

Site fixed effects absorb between-site differences in the level of each construct but not between-site differences in the metric on which it is measured. A supplementary analysis therefore estimated the within-school attitude to perpetration slope separately in each of the 15 education systems and pooled the 15 estimates in a random effects meta-analysis by restricted maximum likelihood. The site-specific slopes were obtained by two independent routes, a single 15-group two-level random slope model and separate two-level models fitted one system at a time, and the two routes agreed to within 0.0006 in every system. Heterogeneity is summarized by Cochran’s Q, the between-site variance component, I squared, and the 95% prediction interval (Higgins et al., 2009). Results appear in Appendix Table A6 and Figure S1.

Because site fixed effects do not absorb between-site differences in measurement, the gender-bias scale was placed on a common metric before the site-specific slopes were interpreted. Two routes were considered. An anchor-item partial-invariance model constrains the loading and the intercept of a small set of items to be equal across systems and frees the remaining item parameters, and it needs items that behave the same way in almost every system. Alignment optimization places all systems on a common metric without anchors, by allowing factor means and variances to differ while minimizing total noninvariance (Asparouhov & Muthen, 2014). Whether the first route was available was assessed from the item-by-system invariance pattern of the alignment solution reported in Table 2, under the conventional requirement that an anchor item hold both its loading and its intercept in at least 13 of the 15 systems. A fully invariant one-factor multiple-group model was fitted for comparison, with maximum likelihood with robust standard errors and schools as clusters. A factor score on the aligned metric was then saved for every student and standardized once in the pooled sample, and the site-specific slopes were re-estimated with that score in place of the OECD index under an unchanged specification. Appendix Table A7 reports the measurement results and Appendix Table A8 reports the re-estimated slopes.

A series of sensitivity analyses, conducted with multilevel models in Stata 18, evaluated the robustness of the focal findings. These analyses re-estimated the cross-level moderation using a leave-one-out school mean of the relational scales, which removes the focal student’s own report from the school aggregate, compared unweighted and sampling weighted specifications, and repeated the model while omitting each site in turn to assess the influence of any single system. Model 3 was also re-estimated separately among boys and among girls, with the same covariate set and site indicators, so that the gender difference implied by the three-way interaction could be evaluated without the constraints that the pooled specification imposes. These split-sample results appear in Appendix Table A4.

## Results

### Descriptive Statistics and Preliminary Analyses

Descriptive statistics for all study variables appear in Table 1. The unconditional model indicated that 11.3% of the variance in bullying perpetration lay between schools (intraclass correlation = 0.113, 95% confidence interval [0.103, 0.124]), which supports the use of a two-level specification. Gender differences in the individual-level variables are summarized as standardized mean differences (SMD) in Table 1. Boys endorsed gender stereotypes more strongly than girls overall (SMD = 0.53), most pronouncedly for the agentic and status subdimension (SMD = 0.84) and least for the communal and care subdimension (SMD = 0.23), and reported more frequent perpetration (SMD = 0.38); SES, acquiescence, and age were balanced across gender, with absolute standardized mean differences of 0.09 or less.

Response distributions for the individual perpetration items are reported in Appendix Table A5. Endorsement of a behavior a few times a year or more often ranged from 11.3% to 27.2% across the six items, and endorsement a few times a month or more often ranged from 4.7% to 9.8%. Boys reported higher endorsement than girls on every item, and the size of the difference tracked the form of the behavior. The ratio of the boys’ percentage to the girls’ percentage was 2.34 for hitting or pushing and 2.30 for taking or destroying belongings, compared with 1.43 for leaving other students out of things and 1.64 for spreading rumors.

### Measurement of the Gender-Bias Scale

The measurement analysis of the gender-bias scale is summarized in Table 2. A single-factor solution fit the eight items poorly, whereas a two-factor solution that separated agentic and status stereotypes from communal and care stereotypes, with an acquiescence covariate, fit the data adequately, chi-square(25) = 5126.38, comparative fit index = 0.943, Tucker-Lewis index = 0.917, root mean square error of approximation = 0.066, 90% confidence interval [0.064, 0.067], standardized root mean square residual = 0.059. Standardized loadings were substantial on both factors, from 0.510 to 0.857 for agentic and status and from 0.729 to 0.754 for communal and care, and the two factors were moderately correlated (*r* = .654). Acquiescence was positively associated with both factors (0.135 and 0.206), which supports adjusting for it in the substantive models.

Turning to the measurement research question, the alignment analysis across the 15 older-cohort sites flagged approximately 48% of intercept and loading parameters as noninvariant. The figures are 50.8% of intercepts and 45.8% of loadings, or 48.3% overall, well above the 25% benchmark for approximate invariance. This pattern indicates that the gender-bias scale does not satisfy cross-site scalar invariance, so latent means were not compared across sites and attitudinal scores were standardized within the analytic sample. These measurement results are reported as a substantive finding about the cross-national comparability of adolescent gender-attitude measures rather than as a nuisance.

Both subscales were reliably measured. In the pooled sample, McDonald’s omega was 0.82 for the agentic and status subscale and 0.83 for the communal and care subscale, and the tau-equivalent coefficient was virtually identical. Estimated separately within each system, omega ranged from 0.69 to 0.86 for agentic and status and from 0.66 to 0.87 for communal and care (Appendix Table A1). Reliability met the conventional 0.70 threshold in every system except Kudus, where both subscales fell in the high .60s, and the communal and care subscale in Delhi. The two subscales are therefore suitable for use across systems.

### Individual and Family Correlates of Perpetration

Results for the substantive models appear in Table 3. In the random-intercept baseline (Model 1), gender-bias attitudes were positively associated with bullying perpetration at the individual level (b = 0.078, SE = 0.006, *p* < .001), and students who endorsed gender stereotypes more strongly reported more frequent perpetration, consistent with Hypothesis 1. The corresponding association at the school level was also positive and larger in magnitude (b = 0.287, SE = 0.051, *p* < .001), indicating that schools whose students held more stereotypical attitudes on average reported more perpetration on average. Coefficients for the individual covariates are reported in Table 3, where girls reported markedly less perpetration than boys (b = − 0.324, SE = 0.010, *p* < .001).

Model 2 added the family predictor and decomposed the association of home female roles with perpetration into a pathway through gender-bias attitudes and a direct pathway. At the individual level, a more female-concentrated division of domestic labor was associated with stronger endorsement of gender stereotypes (a path: b = 0.118, SE = 0.006, *p* < .001), and stronger endorsement of stereotypes was in turn associated with more perpetration (b path: b = 0.068, SE = 0.006, *p* < .001). The within-school indirect association was positive and its confidence interval excluded zero (indirect = 0.008, SE = 0.001, 95% confidence interval [0.006, 0.010]), and a direct association of home female roles with perpetration remained (b = 0.050, SE = 0.005, *p* < .001). At the school level, mean stereotype endorsement was associated with mean perpetration (b path: b = 0.216, SE = 0.037, *p* < .001), and the school-level indirect association was positive and larger than its individual-level counterpart (indirect = 0.175, SE = 0.031, 95% confidence interval [0.114, 0.235]), whereas the school-level direct association of home female roles with perpetration was not significant (b = 0.119, SE = 0.097, *p* = .220). This pattern supports Hypothesis 2 at both levels and indicates that at the school level the association operated primarily through the attitude composite. As stated in the Statistical Analysis, these indirect coefficients decompose covariance and do not establish a temporal or causal sequence from household division of labor through gender-bias attitudes to bullying perpetration.

### School Relational Climate: Level and Slope

**Fig. 1:**
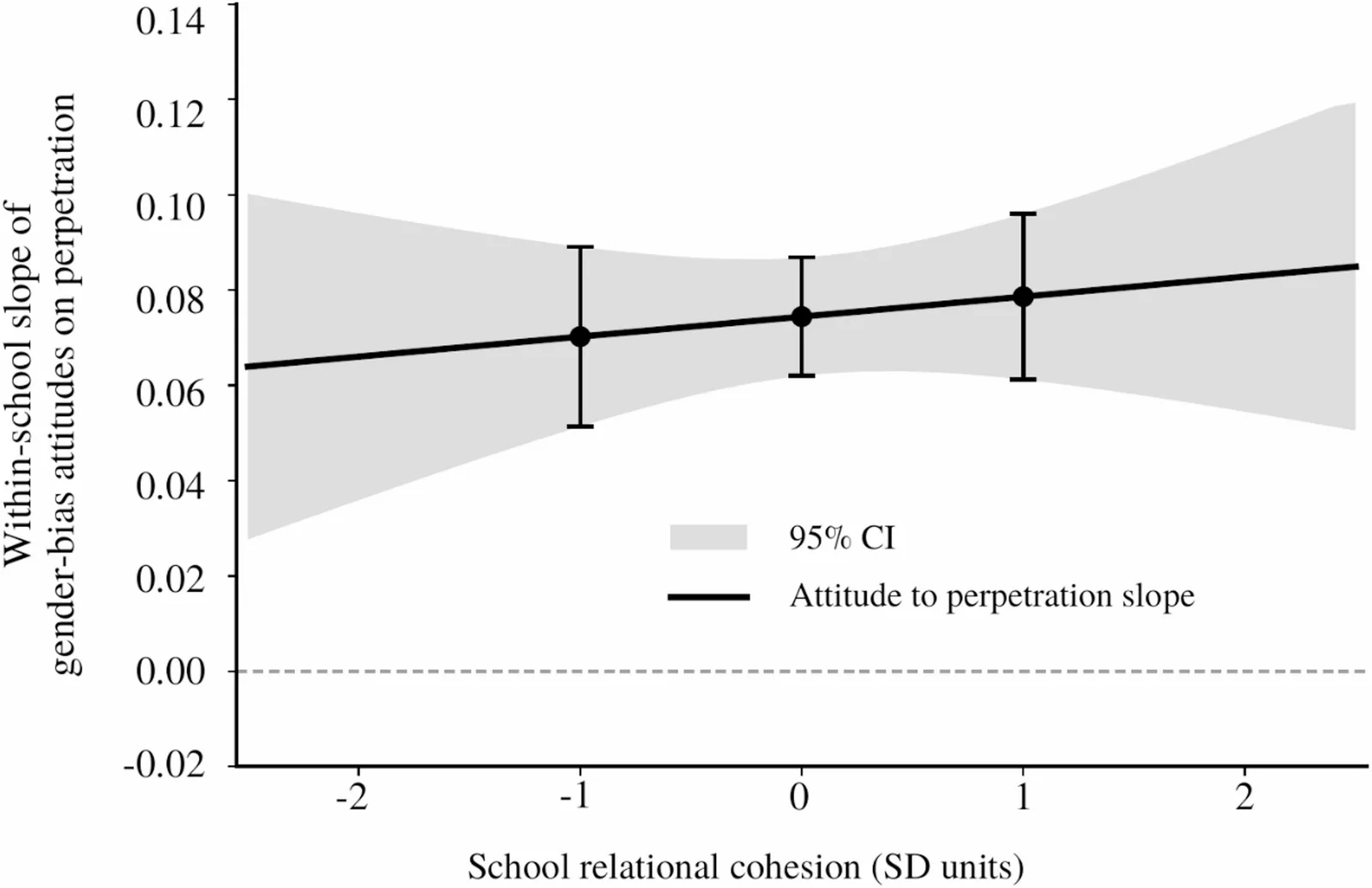
Within-School Slope of Gender-Bias Attitudes on Bullying Perpetration Across Levels of School Relational Cohesion (Model 3)

Model 3 tested whether the strength of the individual attitudes-perpetration association varied with the school’s relational cohesion. The random slope of attitudes on perpetration varied significantly across schools (slope variance = 0.007, *p* < .001), which indicates that the attitude to perpetration association was stronger in some schools than in others. This variation was not explained by relational cohesion, and the cross-level interaction was small and not statistically significant (b = 0.004, SE = 0.007, *p* = .534). The estimated slope of attitudes on perpetration was essentially constant across levels of the moderator, at 0.070 at one standard deviation below the mean, 0.074 at the mean, and 0.079 at one standard deviation above the mean, and its confidence band did not include zero at any level (Fig. 1). The buffering prediction (Hypothesis 4), that a more supportive relational climate would weaken the individual coupling between gender-biased attitudes and perpetration, was therefore not supported in the pooled sample.

At the same time, relational cohesion showed a robust association with perpetration at the school level, consistent with Hypothesis 3. Schools higher in relational cohesion reported less perpetration on average (b = − 0.101, SE = 0.010, *p* < .001), with adjustment for the school-level attitude composite, school composition, and site fixed effects. School institutional order, which indexes formal social control, was not associated with perpetration (b = 0.008, SE = 0.008, *p* = .335). Higher school mean SES was associated with slightly more perpetration (b = 0.028, SE = 0.014, *p* = .042), and a higher proportion of female students was associated with slightly less perpetration (b = − 0.020, SE = 0.008, *p* = .007). The relational climate of the school was thus associated with the overall level of perpetration even though it did not moderate the individual attitude to perpetration association.

Model 4 examined whether the conditional association differed for girls and boys by adding a within-level product of female and attitudes together with its cross-level moderation. The three-way interaction of relational cohesion, attitudes, and female reached the conventional significance threshold (b = − 0.025, SE = 0.012, *p* = .046), which suggests that the way relational climate related to the attitude to perpetration slope differed by gender, in the direction anticipated by Hypothesis 5. Simple slopes describe the pattern (Figure S3 and Appendix Table A3). Among boys, the attitude to perpetration slope was larger in schools higher in relational cohesion, rising from 0.089 at one standard deviation below the mean to 0.125 at one standard deviation above. Among girls, the slope was smaller in schools higher in relational cohesion, falling from 0.058 to 0.045 across the same range. The attitude to perpetration slope was positive for both genders at every level of the moderator, so the difference is one of degree and not of direction. This interaction is treated as exploratory throughout. Its confidence interval reaches zero at one bound, the sampling weighted specification did not reproduce it at the conventional threshold, and the split-sample models reported below did not reproduce it as a reliable effect. No substantive claim in this article rests on it.

### Site-Specific Slopes

The within-school slope of perpetration on gender-bias attitudes was estimated separately in each education system and pooled by random effects meta-analysis (Appendix Table A6 and Figure S1). Estimates ranged from − 0.048 in Bulgaria to 0.151 in Sobral. Fourteen of the 15 confidence intervals lay entirely above zero, and the exception was Bulgaria, whose slope was negative with an interval that included zero (b = − 0.048, 95% confidence interval [-0.117, 0.021]). Random effects pooling gave b = 0.080, SE = 0.010, 95% confidence interval [0.059, 0.100], z = 7.67, *p* < .001, close both to the common effect estimate (b = 0.074, 95% confidence interval [0.064, 0.085]) and to the pooled within-site coefficient obtained under site fixed effects in Model 3. Between-site heterogeneity was substantial, Q(14) = 46.08, *p* < .001, with a between-site variance component of 0.0011, I squared of 71.0%, and H squared of 3.45, and the 95% prediction interval was [0.006, 0.153]. The direction of the association was positive in 14 of the 15 systems while its magnitude varied.

Whether the gender-bias scale could be placed on a common metric was examined first (Appendix Table A7). No item met the conventional anchor criterion. The item that came closest was sensitivity, which held both its loading and its intercept in 6 of the 15 systems, and no other item held both in more than 4. Noninvariance was concentrated in particular parts of the scale. The loadings of the three agentic and status items carried invariance indices of 0.122 for ambition, 0.050 for leadership, and 0.095 for technology, and the intercept of the ambition item carried an index of 0.000. The empathy intercept and the aggressiveness loading were close to invariant, at 0.960 and 0.840. The number of flagged parameters varied widely by system, from 2 of 16 in Turin and in Emilia-Romagna to 12 of 16 in Bogota. A fully invariant one-factor multiple-group model fitted the data poorly, with chi-square(496) = 33,831.29, a comparative fit index of 0.699, a Tucker-Lewis index of 0.745, a root mean square error of approximation of 0.146, 90% confidence interval [0.145, 0.148], and a standardized root mean square residual of 0.131. An anchored common metric is therefore not available for this scale in these systems, and alignment was used to harmonize the measure.

The 15 site-specific slopes were then re-estimated with the aligned score in place of the OECD index (Appendix Table A8 and Figure S2). The two versions of the attitude measure correlated 0.947 at the student level. The 15 slope estimates correlated 0.90 across metrics, and their rank correlation was 0.89. A site slope moved by 0.004 on average, with a standard deviation of 0.027 and a range from − 0.047 to 0.051. Five systems moved by more than 0.02, namely Delhi (+ 0.051), Dubai (+ 0.044), Emilia-Romagna (+ 0.030), Bulgaria (-0.047), and Sobral (-0.047).

Pooling the aligned estimates gave b = 0.083, SE = 0.014, 95% confidence interval [0.056, 0.111], z = 5.92, *p* < .001. The same models on the OECD index gave b = 0.077, SE = 0.010, 95% confidence interval [0.057, 0.097], z = 7.52, *p* < .001. Harmonizing the measure left the pooled association almost unchanged and widened the dispersion around it. On the aligned metric Q(14) was 70.45, *p* < .001, the between-site variance component was 0.0024, I squared was 83.7%, and H squared was 6.13. The corresponding values on the OECD index were Q(14) = 42.77, *p* < .001, a variance component of 0.0010, I squared of 68.4%, and H squared of 3.16. The 95% prediction interval widened from [0.006, 0.148] to [-0.026, 0.193]. Fourteen of the 15 confidence intervals lay entirely above zero on the aligned metric. The exception was Bulgaria at − 0.093, 95% confidence interval [-0.162, − 0.025], which is reliably negative once the measure is harmonized.

### Gender Violence Climate and School Composition

Model 5, estimated among older-cohort schools, added school gender-violence climate and retained the composition covariates. The school-level association of relational cohesion with perpetration remained negative and significant after these additions (b = − 0.066, SE = 0.012, *p* < .001), which indicates that this association was not accounted for by the demographic composition of the school or by its gender climate. School gender violence climate was itself positively associated with perpetration at the school level (b = 0.079, SE = 0.015, *p* < .001), so that in schools where students perceived more gender-based harassment and disrespect, reported perpetration was higher, consistent with Hypothesis 6. Gender violence climate was also associated with a somewhat steeper attitude to perpetration slope (b = 0.016, SE = 0.007, *p* = .025), whereas the cross-level moderation by relational cohesion again did not reach significance (b = 0.013, SE = 0.009, *p* = .131). The school-level association of gender-bias attitudes with perpetration was attenuated to nonsignificance once gender-violence climate was included (b = 0.071, SE = 0.055, *p* = .197), which indicates that the school-level attitude composite and the gender climate construct share variance in their relation to perpetration.

### Moderated Mediation

**Fig. 2:**
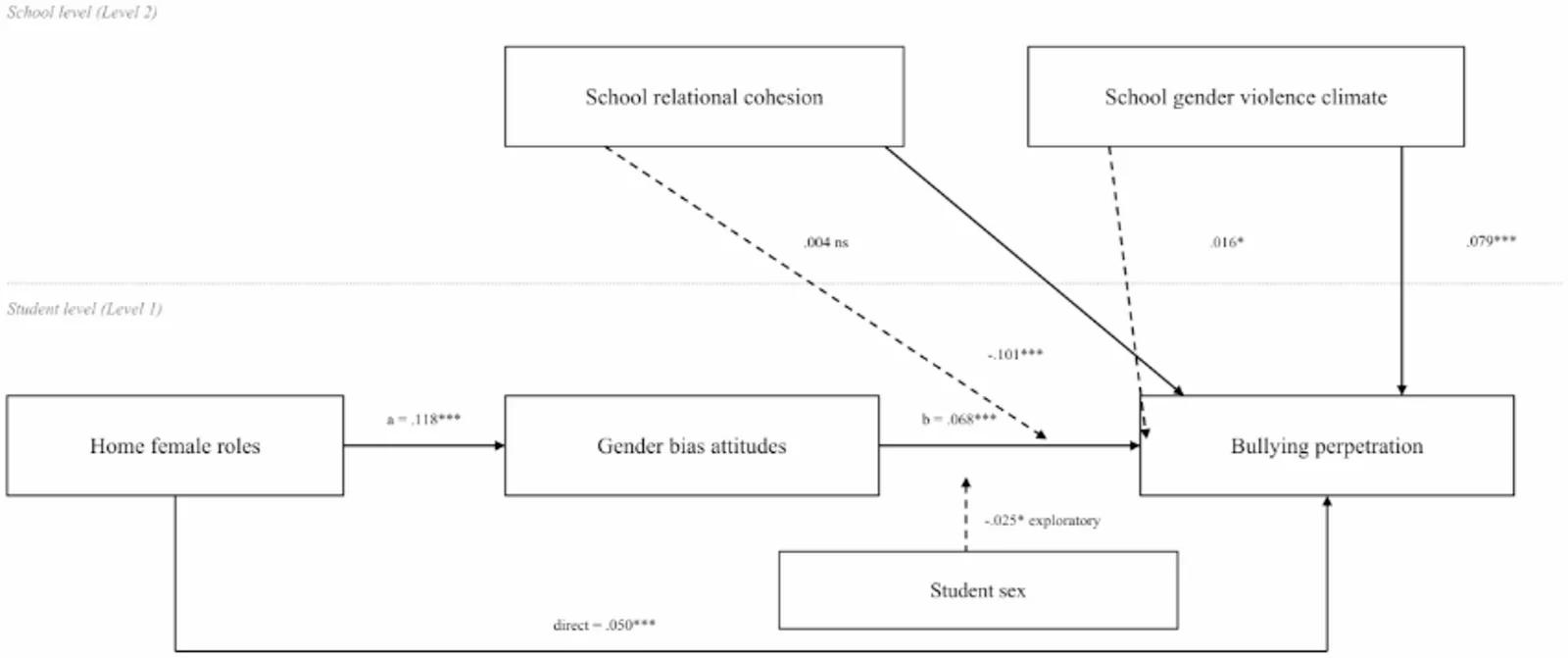
Summary of the Estimated Model

Model 6 re-estimated the mediation pathway with the attitude to perpetration slope moderated by relational cohesion, using Bayesian estimation (Table 4). The individual-level a path from home female roles to attitudes and the mean b path from attitudes to perpetration were both positive and credibly different from zero, with a = 0.122, 95% credible interval [0.112, 0.132], and a mean slope of 0.068, 95% credible interval [0.055, 0.080], as was the direct association of home female roles with perpetration, 0.053, 95% credible interval [0.043, 0.063]. The conditional indirect association of home female roles with perpetration through attitudes was positive and credible at every level of the moderator, and it varied only trivially across levels, from 0.007 at one standard deviation below the mean, to 0.008 at the mean, to 0.009 at one standard deviation above the mean. The index of moderated mediation was small and its 95% credible interval reached zero at its lower bound (index = 0.002, 95% credible interval [0.000, 0.006]), which indicates that the indirect pathway did not credibly differ across levels of relational cohesion. This result is consistent with the nonsignificant cross-level moderation in Models 3 and 5. The school-level association of relational cohesion with perpetration was again negative and credible (b = − 0.104, 95% credible interval [-0.122, − 0.086]). The estimated model is summarized in Fig. 2.

### Sensitivity Analyses

The focal findings were stable across the sensitivity analyses conducted in Stata. When the school mean of the relational scales was recomputed with the focal student’s own report removed, the school-level association of relational cohesion with perpetration remained negative and significant, and a small positive cross-level interaction emerged (b = 0.015, SE = 0.005, *p* = .004). In the sampling weighted specification, the coefficients retained their sign and significance, and the cross-level interaction remained nonsignificant (b = 0.025, SE = 0.013, *p* = .058). Repeating the model while omitting each site in turn produced cross-level interaction coefficients that ranged narrowly from 0.003 to 0.015, none of which altered the substantive conclusions. Across all specifications, the positive individual-level association between gender-bias attitudes and perpetration and the negative school-level association between relational cohesion and perpetration were consistent, whereas the cross-level moderation of the individual association by relational cohesion was consistently small. Aggregating the relational scales to the school level was well supported, since in unconditional two-level models the intraclass correlation ICC(1) ranged from 0.068 to 0.092 and ICC(2) for the three relational scales and the composite ranged from 0.73 to 0.79 at the average school size, all meeting conventional thresholds for aggregation (Appendix Table A2).

Model 3 was also re-estimated separately among boys (*n* = 19,063 in 1,105 schools) and among girls (*n* = 19,135 in 1,104 schools), with the same covariate set and site indicators (Appendix Table A4). School relational cohesion was associated with a lower school mean level of perpetration in both subsamples (boys b = − 0.130, SE = 0.014, 95% confidence interval [-0.157, − 0.104]; girls b = − 0.096, SE = 0.012, 95% confidence interval [-0.119, − 0.073]). The mean within-school slope of perpetration on gender-bias attitudes was positive in both subsamples and about twice as steep among boys (b = 0.101, SE = 0.010, 95% confidence interval [0.082, 0.120]) as among girls (b = 0.056, SE = 0.008, 95% confidence interval [0.040, 0.071]), with a difference of 0.045, 95% confidence interval [0.020, 0.070]. Cross-level moderation of that slope by relational cohesion was not distinguishable from zero in either subsample (boys b = 0.018, SE = 0.011, 95% confidence interval [-0.004, 0.039]; girls b = − 0.005, SE = 0.008, 95% confidence interval [-0.020, 0.009]), and the two subsample estimates did not differ reliably from each other (difference = 0.023, 95% confidence interval [-0.004, 0.050]). Evaluated at one standard deviation below the mean, at the mean, and at one standard deviation above the mean of relational cohesion, the attitude to perpetration slope moved from 0.083 to 0.101 to 0.119 among boys and from 0.061 to 0.056 to 0.050 among girls, and neither trajectory departed reliably from a flat line (boys, change across two standard deviations = 0.036, 95% confidence interval [-0.007, 0.079]; girls, − 0.010, 95% confidence interval [-0.041, 0.021]). The split-sample estimates therefore reproduce the direction, but not the statistical reliability, of the three-way interaction obtained in the pooled sample.

## Discussion

A supportive school could reduce bullying in either of two ways, by lowering how much bullying occurs or by loosening the connection between what students believe about gender and how they treat one another. The school climate and collective efficacy literatures have not separated these possibilities, even though they point to different prevention strategies. Testing the distinction requires a design in which level and slope are estimated in the same model, and a set of contexts diverse enough to show whether any regularity holds beyond a single system. Mid-adolescence is an informative stage for this test, because peer status and gender conformity are both especially consequential at that age. The present analyses drew on 47,962 mid-adolescents in 1,263 schools across 15 education systems and returned a consistent and bounded answer. Supportive relationships were associated with a lower level of bullying and not with a weaker coupling between belief and behavior, whereas a more hostile gender climate was associated with more bullying and with a modestly tighter coupling.

### The Family, Attitudes, and Perpetration Sequence Across Education Systems

The pathway from household gender arrangements through adolescents’ own gender attitudes to their bullying of peers held within schools, and a direct association of household labor division with perpetration remained after the attitude pathway was taken into account. Two features of the family measure deserve more weight than the earlier framing gave them. First, the indirect component is small in absolute terms while the direct component is of similar magnitude, which suggests that household arrangements are associated with adolescent bullying through channels beyond the stereotype endorsement measured here. Candidate channels include the modeling of power relations within the household, exposure to gendered role prescriptions that operate without being endorsed as beliefs, and the family conflict that unequal domestic arrangements can accompany. Second, the association was visible at the school level as well as at the student level, which is the level at which school-based prevention would operate. This extends work conducted at the individual level in the same survey (Zheng et al., 2026) and converges with the developmental and sociological literature on how children read the gender arrangements they observe at home (Coltrane, 1989; Croft et al., 2014). It also supports the framing of adolescent bullying as an early expression of the same belief system implicated in adult intimate partner violence (Jewkes et al., 2015; Reyes et al., 2016), across a set of education systems considerably more diverse than the North American and Western European samples that dominate this literature (Kagesten et al., 2016).

The indirect association of household gender arrangements with perpetration through attitudes was appreciably larger between schools than within schools. This descriptive contrast should not be read as evidence of an ecological process. A between-school coefficient absorbs, in addition to any substantive process, the selection of families into schools with particular gender profiles, shared reporting styles among students in the same school, and unmeasured features of local communities that shape both household arrangements and behavioral norms. Establishing whether household arrangements produce school-level differences in gender attitudes, and thereby in bullying, requires longitudinal and multi-informant designs in which household composition is measured before student attitudes and student attitudes are measured before behavior, most cleanly at the classroom level, where the peer processes theorized here operate.

### A Protective Association Without Buffering

School relational cohesion was consistently associated with less perpetration at the school level, and this association survived adjustment for school mean socioeconomic status, gender composition, immigrant density, site, and gender violence climate. It did not flatten the within-school attitude to perpetration slope. The estimated slope was essentially constant across the range of the moderator, the cross-level interaction was small with a confidence interval tightly bracketing zero, and the index of moderated mediation was likewise not credibly different from zero. With 38,198 students in 1,163 schools, this is a precise null and not an underpowered one, since the data are informative about the absence of a buffering association of any substantively meaningful size.

This pattern should be read narrowly. The analysis shows that a relational cohesion measure, built from belonging, relationships with classmates, and relationships with teachers aggregated to the school level, is associated with a lower level of bullying without weakening the within-person coupling between belief and behavior. It does not establish whether collective efficacy in the original sense would buffer that coupling, because the intervention component of collective efficacy, meaning the willingness of members to sanction aggression, was not directly measured. A composite of principal-reported school safety and anti-bullying provision, which captures formal social control, likewise did not moderate the attitude to perpetration slope, so neither of the two control-related measures available here bore on the slope. A direct student report of collective willingness to intervene against peer aggression, which the Survey on Social and Emotional Skills 2023 does not include, might behave differently. The findings are therefore consistent with two readings. Relational cohesion on its own may not be the aspect of the school context that changes the process by which beliefs are enacted. Alternatively, cohesion and willingness to intervene may both be needed to change that process, so that one component acting alone cannot. Distinguishing these readings requires the direct measure of informal social control that this study, like most school climate work to date, lacks.

The null result for buffering is nonetheless informative for theory. Collective efficacy, as originally formulated, is an account of rates, of why some collectivities have less violence than others given their composition (Sampson et al., 1997). It is not, in its original form, an account of the within-person coupling between belief and behavior. Classroom norm research points in the same direction, with norms accounting for group-level variance while individual attitudes continue to predict individual behavior (Saarento & Salmivalli, 2015). The practical reading is that improving school relationships is worth doing on its own terms, and that it is unlikely to be sufficient for addressing gender-inequitable beliefs, which appear to require direct attention (Gaffney et al. 2021a).

### Gender Asymmetry in How the School Context Operates

The pooled three-way interaction of relational cohesion, attitudes, and gender pointed to a divergence between girls and boys. Among girls, the attitude to perpetration slope was smaller in schools higher in relational cohesion, and among boys it was larger, with the slope remaining positive for both groups at every level of the moderator. When boys and girls were modeled separately, this divergence was not reproduced as a reliable effect. Cross-level moderation of the slope by relational cohesion was positive among boys and negative among girls, as the interaction implies, yet neither coefficient was distinguishable from zero and the two did not differ reliably from each other (Appendix Table A4). Whether more cohesive schools weaken the belief to behavior link differently for boys and for girls therefore remains open, and the three-way interaction is best read as a hypothesis for replication in samples designed to test it.

Two findings were reproduced when boys and girls were modeled separately, and they carry more weight than the three-way interaction. The first is the asymmetry between level and slope, since relational cohesion was associated with a lower school mean level of perpetration among boys and among girls alike, while the within-school attitude to perpetration link was not detectably weaker in more cohesive schools in either group. The second concerns the link itself and not its moderation, in that the slope was about twice as steep among boys as among girls. Part of that difference may reflect what the outcome index captures best. Boys reported higher endorsement than girls on every perpetration item, and the size of the gap tracked the form of the behavior. On the three direct items, covering physical aggression, threats, and property, boys’ endorsement was about 2.3 times girls’, compared with about 1.5 times on the two relationally oriented items, covering exclusion and rumor spreading (Appendix Table A5). A composite that weights six items equally, three of which describe direct aggression, will be more sensitive to variation in boys’ behavior than in girls’. The steeper slope among boys may therefore reflect, in part, the behavioral content the index captures best and not only a difference in how attitudes translate into conduct.

The mechanisms sketched in the introduction remain plausible accounts of a gender difference in contextual moderation, though none was tested here. Girls’ aggression is more often relational in form and therefore more embedded in the ties that a cohesive school makes dense and visible, so relational monitoring is more likely to reach it (Card et al., 2008; Rose & Rudolph, 2006), whereas boys’ aggression is more closely tied to public status competition, for which a densely connected school supplies a larger and more attentive audience (Reigeluth & Addis, 2016). Any such dynamic would be expected to be pronounced at the age studied here, because the mid-adolescent years are when popularity motives most strongly drive perpetration (Romera et al., 2021). Contexts that look healthier can carry counterintuitive conditional effects for particular subgroups, as documented under the heading of the healthy context paradox (Garandeau & Salmivalli, 2019). A methodological lesson also follows and does not depend on the effect surviving replication. When a contextual effect is expected to operate in opposite directions for two groups, a pooled cross-level interaction can return a well-estimated zero that describes no one, so gender-disaggregated moderation deserves to be the default analysis in this literature and not a supplementary one.

### Hostile Climate as an Active Risk

Schools in which students perceived more gender-based harassment and disrespect reported more perpetration, with adjustment for composition and for relational climate, and this hostile climate was also associated with a somewhat steeper attitude to perpetration slope. The presence of gender hostility and the absence of relational cohesion are not the same variable with opposite signs. Both entered the model, both were associated with the level of perpetration, and only the hostile climate showed any association with the slope. Once gender violence climate was included, the school-level association of aggregate gender-bias attitudes with perpetration was attenuated to nonsignificance, which indicates that the school-level attitudinal composite and the perceived climate share substantial variance in their relation to perpetration. This is consistent with recent cross-national evidence that school-based gender-based violence is associated with bullying perpetration and that the association is stronger in higher-masculinity cultural contexts (Jiang et al., 2026), and with multi-informant work showing that school climate conditions the prevalence of gendered harassment (Rinehart & Espelage, 2016).

The policy implication of the hostile climate finding is more specific than a general call for warmer schools. Interventions that raise cohesion without addressing gender hostility target a variable associated with the level of bullying but not with the strength of the belief to behavior link, whereas interventions that reduce gender-based harassment target a variable associated with both. Programs that combine relational support with explicit critical work on gender norms, such as coach-delivered gender violence prevention with adolescent boys (Miller et al., 2020) and gender-transformative programming more broadly (Amin et al., 2018), are better matched to this pattern than either component alone. Whole-school anti-bullying programs are effective on average (Gaffney et al. 2021b), and component analyses indicate that what programs contain matters as much as whether they exist (Gaffney et al. 2021a). The present results suggest that it is their gender-specific content, and not their relational content, that would be expected to bear on the attitude to behavior coupling.

### Implications for the Measurement of Gender Attitudes

Alignment analysis flagged approximately 48% of the intercept and loading parameters of the gender-bias scale as noninvariant across the 15 sites, roughly twice the conventional benchmark for approximate invariance (Asparouhov & Muthen, 2014). A single-factor solution also fit poorly, whereas a two-factor solution distinguishing agentic and status stereotypes from communal and care stereotypes fit adequately once acquiescent responding was controlled. Substantively, this indicates that adolescents’ gender stereotyping is not a single dimension. Beliefs about who leads and who is aggressive are separable from beliefs about who cares and who is sensitive, and the two need not move together across contexts. Methodologically, latent means on this scale should not be compared across education systems, so cross-national prevalence rankings and league tables of how traditional adolescents are in different sites are not warranted on this instrument (Putnick & Bornstein, 2016).

The noninvariance of the gender-bias scale bears on cross-national comparisons of associations as well as of levels. Site fixed effects absorb differences in level, and they do not absorb differences in the metric on which a construct is measured, so a pooled within-site coefficient can in principle average over relationships that are not substantively the same. Estimating the attitude to perpetration slope separately in each of the 15 systems and pooling the estimates addresses part of this concern (Appendix Table A6). The pooled slope was close to the coefficient obtained under site fixed effects, so the pooled result is not an artifact of any single system, and the association was positive in 14 of the 15 systems.

The magnitude of the attitude to perpetration association, unlike its direction, varied substantially across education systems. Site-specific slopes ranged from a nonsignificant negative value in one system to about twice the pooled value in another, and most of that variation exceeded what sampling error alone would produce. The prediction interval indicates that the slope in a new comparable system could fall anywhere from negligible to nearly twice the pooled estimate. The association between gender-inequitable attitudes and bullying perpetration is therefore best described as directionally general and quantitatively local.

Whether the variation in the slope across systems is substantive or an artifact of measurement noninvariance can be answered with the harmonized metric. No item in this scale behaved consistently enough across systems to anchor a common metric, and a model imposing a common metric by constraint on every item fitted the data poorly (Appendix Table A7), so alignment was used instead. Re-estimating the site-specific slopes on the aligned metric left the pooled association almost unchanged and made the variation between systems larger rather than smaller, with the prediction interval widening to include zero (Appendix Table A8). Cross-system variation in the strength of the belief to behavior link is therefore not an artifact of the noninvariance documented here. Bulgaria makes the point concrete. Its slope was negative and imprecise on the original index and became reliably negative once the measure was harmonized, so a scale that travels badly can obscure a real reversal instead of manufacturing one.

### Limitations

Five features bound what these results can support. The data are cross-sectional and were collected for another purpose, so the indirect pathway is a decomposition of covariation rather than evidence of a temporal sequence, and reverse and reciprocal pathways are equally plausible. Because both the outcome and the principal predictors are student self-reports collected in a single instrument, common method variance and social desirability may inflate the within-level associations. Adjustment for acquiescent responding attenuates but does not eliminate this concern, and although a leave-one-out specification reproduced the school-level association, shared perceptual bias within schools cannot be excluded. The analytic units are schools rather than classrooms, although much of the peer group process theorized here operates at the classroom level (Salmivalli & Voeten, 2004), which may attenuate contextual associations. The participating sites are cities and sub-national regions as well as countries, and the samples are not nationally representative. The outcome measure constrains the theoretical claim in two further respects. The index is best understood as the frequency of aggressive conduct toward peers, since responses are anchored on how often each behavior occurred across the school year, so that the two higher response categories carry the repetition requirement that classic definitions impose, while the scale contains no direct measure of the power imbalance criterion (Olweus, 1993). It is also a general composite of relational, verbal, and physical items that does not separately identify gender-based harassment, homophobic teasing, reputational aggression, or aggression directed toward gender-nonconforming peers. The findings are therefore consistent with the framing of adolescent bullying as an arena in which gendered belief systems are enacted, and they do not demonstrate it. A more direct test would require type-specific outcomes, ideally reported by peers rather than by potential perpetrators.

Two features of the sample warrant particular caution. Gender was operationalized as a binary variable from students’ own reports, and the 552 students, or 1.2% of the sample, who reported another category or declined to answer were excluded from the analytic models. Adolescents whose gender identity or expression departs from binary expectations are at elevated risk of gender-based harassment, and excluding them from a study about the enforcement of gender norms removes from view the group for whom that enforcement may be most consequential (Rinehart & Espelage, 2016). The older cohort also spans only about 14 months around age 15, so whether a level association without a slope association also characterizes early adolescence, when gender attitudes are less consolidated, remains open.

### Implications and Future Directions

These results suggest a division of labor in school-based prevention. Building supportive relationships is associated with less bullying overall and is worth doing on those grounds, and it should not be expected to dissolve the association between gender-inequitable beliefs and aggressive behavior, which persisted at full strength in the most cohesive schools in the sample. A less hostile gender climate was associated both with less perpetration and, modestly, with a weaker belief to behavior coupling, which makes gender hostility the more promising target for reducing the behavioral expression of gendered beliefs. Because the family arrangements associated with these beliefs were visible at the school level as well as the individual level, prevention that stops at the school gate is unlikely to reach the whole of the process.

Several classes of factors beyond the school relational climate may weaken the belief to behavior link and deserve systematic testing. Social and emotional skills such as self-control and empathy could plausibly attenuate the translation of hostile beliefs into hostile behavior, moral disengagement could amplify it, and dyadic positive peer relationships that are invisible to school-mean measures may operate protectively at a level this design cannot see. The most direct candidates are programs that target normative beliefs at the point of behavior, such as gender-transformative curricula and coach-delivered gender violence prevention (Amin et al., 2018; Miller et al., 2020). Whether such programs lower only the level of bullying, or also weaken the attitude to behavior slope, is the same question raised here and is a natural target for the next generation of trials in mid-adolescence.

Four lines of methodological work would strengthen the evidence on which these conclusions rest. Longitudinal and multi-informant designs are needed to establish temporal order and to reduce shared-method bias, ideally with peer or teacher reports of perpetration. Analyses at the classroom level, and of gender composition within classrooms, would test whether the asymmetry reported here is a property of the immediate peer group. A direct student report of collective willingness to intervene against peer aggression, combined with a cohesion measure, would allow the intervention component of collective efficacy to be tested properly, which the present data cannot do. Measurement work on gender attitudes in non-Western adolescent populations, developing and validating locally grounded instruments and reporting invariance evidence as a matter of routine, would allow the cross-national comparisons that the current generation of instruments cannot support. Harmonizing an existing scale after the fact does not remove that need. No item in this scale was stable enough to anchor a common metric across these systems, and the alignment-based harmonization that the data did support left the variation between systems larger than before.

## Conclusion

By mid-adolescence, beliefs about what boys and girls should be are largely formed, and schools are where those beliefs meet the peer group. Whether a warmer school changes how bullying happens, or only how often, has not been separated in this literature, and the two answers imply different prevention strategies. Data from 47,962 mid-adolescents in 1,263 schools across 15 education systems showed that the distinction is real. Supportive school relationships were associated with less bullying overall and did not weaken the coupling between gender-inequitable beliefs and aggressive behavior, whereas a more hostile school gender climate was associated with more bullying and with a modestly tighter coupling. The gendered organization of household labor was associated with perpetration through adolescents’ own attitudes at the student level and at the school level, so the beliefs at issue are not formed inside the school alone. An exploratory difference between girls and boys in how cohesive schools relate to the belief to behavior slope was not reproduced in split-sample models and awaits replication. For adolescents around age 15, when peer status and gender conformity are both especially consequential, warm relationships and the reduction of gender hostility are therefore distinct targets, since the first was associated with the level of bullying alone and only the second was also associated with the strength of the link between beliefs and behavior. Effective prevention at this age will require both, and it will require reaching beyond the school gate to the household arrangements in which these beliefs are learned. A final caution follows from the measurement work, since a widely fielded gender attitude scale did not meet the approximate invariance criterion across systems, which places a limit on the cross-national comparisons that this generation of instruments can support.

## Supplementary Information

Below is the link to the electronic supplementary material.

Supplementary Material 1

## Data Availability

The data analyzed in this study are from the OECD Survey on Social and Emotional Skills 2023, older cohort. The questionnaires and code-books are publicly available at https://www.oecd.org/en/data/datasets/SSES-Round-2-Database.html, and access to the microdata is granted by the OECD on application through that page. Analysis syntax is available from the author on reasonable request.
