# Supplementary material for: School Relational Cohesion, Gender-inequitable Attitudes, and Bullying Perpetration Across 15 Education Systems: Less Bullying Overall Without a Weaker Attitude-behavior Link
Source: J Youth Adolesc. 2026 Sep 24;55(10):2507–27. doi: 10.1007/s10964-026-02416-8 (PMC13630974; doi:10.1007/s10964-026-02416-8)
Supplement: Supplementary file 1 [file 10964_2026_2416_MOESM1_ESM.docx]

**Appendix**

**Supplementary Tables**

**Table A1 Internal Consistency of the Gender-Bias Subscales, Overall and by Education System**

| **System** | **n** | **ω agentic** | **α agentic** | **ω communal** | **α communal** |
| --- | --- | --- | --- | --- | --- |
| Pooled | 47,165 | .817 | — | .832 | — |
| Bogotá (Colombia) | 3,722 | .850 | .847 | .857 | .853 |
| Bulgaria | 2,919 | .788 | .793 | .821 | .822 |
| Chile | 3,575 | .833 | .831 | .838 | .837 |
| Delhi (India) | 2,624 | .751 | .754 | .672 | .672 |
| Dubai (United Arab Emirates) | 5,571 | .856 | .869 | .806 | .808 |
| Gunma (Japan) | 3,469 | .795 | .794 | .871 | .871 |
| Helsinki (Finland) | 2,364 | .838 | .839 | .850 | .850 |
| Jinan (China) | 3,273 | .846 | .852 | .820 | .821 |
| Kudus (Indonesia) | 3,448 | .691 | .687 | .663 | .654 |
| Peru | 2,798 | .759 | .755 | .811 | .805 |
| Sobral (Brazil) | 2,290 | .736 | .726 | .808 | .806 |
| Spain | 3,278 | .833 | .839 | .835 | .834 |
| Turin (Italy) | 3,008 | .839 | .845 | .833 | .829 |
| Emilia-Romagna (Italy) | 2,970 | .825 | .834 | .810 | .807 |
| Ukraine (19 of 27 regions) | 1,856 | .818 | .816 | .823 | .822 |

*Note.* ω = McDonald’s omega from a congeneric two-factor CFA (factor variances fixed to 1, all loadings free). α = model-based reliability under equal loadings (essentially tau-equivalent), the model equivalent of Cronbach’s alpha. “Pooled” = a single two-factor model on all systems with cluster-robust SEs. Systems are listed by name; Mexico is not included, for the reasons given in the Methods.

**Table A2 Aggregation Reliability (Intraclass Correlations) for School-Level Measures**

| **School-level measure** | **ICC(1)** | **ICC(2)** | **95% CI (ICC(2))** |
| --- | --- | --- | --- |
| Belonging | .068 | .729 | [.695, .763] |
| Classmate relations | .082 | .769 | [.741, .797] |
| Teacher-student relations | .089 | .783 | [.758, .808] |
| Relational composite | .092 | .789 | [.764, .815] |
| Bullying perpetration (benchmark) | .113 | .825 | [.800, .850] |

*Note.* Estimates from an unconditional two-level model (N = 46,802 students in 1,262 schools). ICC(1) = between-school share of variance; ICC(2) = reliability of the school mean = (k × ICC(1)) / [1 + (k - 1) × ICC(1)] at the average cluster size k = 37. Bullying perpetration is shown only as a benchmark. ICC(2) ≥ .70 indicates reliable aggregation (Bliese, 2000).

**Table A3 Simple Slopes, Conditional and Indirect Effects (95% CI)**

| **Quantity** | **Estimate** | **95% CI** |
| --- | --- | --- |
| ***M2. Multilevel mediation (home roles → attitudes → perpetration)*** | | |
| Within indirect effect (a×b) | 0.008*** | [0.006, 0.010] |
| Between indirect effect (a×b) | 0.175*** | [0.114, 0.235] |
| ***M3. Attitude-perpetration slope by school relational cohesion*** | | |
| At -1 SD | 0.070*** | [0.051, 0.089] |
| At mean | 0.074*** | [0.062, 0.087] |
| At +1 SD | 0.079*** | [0.061, 0.096] |
| Moderation (slope on school relational cohesion, b1) | 0.004 | [-0.009, 0.017] |
| ***M4. Slopes by gender × school relational cohesion*** | | |
| Boys, -1 SD | 0.089*** | [0.060, 0.117] |
| Boys, +1 SD | 0.125*** | [0.100, 0.150] |
| Girls, -1 SD | 0.058*** | [0.035, 0.080] |
| Girls, +1 SD | 0.045*** | [0.029, 0.060] |
| Boys’ moderation (b1) | 0.018† | [-0.002, 0.038] |
| Girls’ additional moderation (f1) | -0.025* | [-0.049, 0.000] |
| ***M5. Slope by school relational cohesion, net of composition and climate*** | | |
| At -1 SD | 0.062*** | [0.041, 0.082] |
| At +1 SD | 0.088*** | [0.066, 0.110] |

*Note.* Delta-method CIs from MODEL CONSTRAINT (multilevel models do not support bootstrapping); Monte Carlo CIs can be computed from the TECH3 parameter covariance matrices. ±1 SD refers to standardized school relational cohesion. † p < .10. * p < .05. ** p < .01. *** p < .001.

**Table A4 Split-Sample Estimates of Model 3 Among Boys and Among Girls**

|  | **Boys** | | | **Girls** | | | **Difference (boys − girls)** | |
| --- | --- | --- | --- | --- | --- | --- | --- | --- |
| *Parameter* | *b* | *SE* | 95% CI | *b* | *SE* | 95% CI | Δb [95% CI] | *p* |
| *Panel A. Within (student) level — outcome: bullying perpetration* | | | | | | | | |
| Socioeconomic status | 0.055 | 0.012 | [0.032, 0.078] | 0.028 | 0.010 | [0.010, 0.047] | 0.027 [−0.004, 0.058] | .084 |
| Immigrant background | 0.061 | 0.033 | [−0.004, 0.126] | 0.034 | 0.029 | [−0.024, 0.091] | 0.027 [−0.059, 0.113] | .539 |
| Home language other than test language | 0.122 | 0.024 | [0.075, 0.169] | 0.062 | 0.017 | [0.029, 0.096] | 0.060 [0.002, 0.118] | .041 |
| Age | −0.007 | 0.008 | [−0.022, 0.008] | 0.000 | 0.006 | [−0.013, 0.012] | −0.007 [−0.027, 0.013] | .484 |
| Acquiescent response style | 0.047 | 0.010 | [0.027, 0.067] | 0.029 | 0.009 | [0.011, 0.047] | 0.018 [−0.008, 0.044] | .181 |
| Residual variance | 0.967 | 0.018 | [0.931, 1.003] | 0.608 | 0.017 | [0.575, 0.641] | 0.359 [0.310, 0.408] | < .001 |
| *Panel B. Between (school) level — outcome: school mean perpetration* | | | | | | | | |
| School relational cohesion | −0.130 | 0.014 | [−0.157, −0.104] | −0.096 | 0.012 | [−0.119, −0.073] | −0.034 [−0.070, 0.002] | .065 |
| School institutional order | 0.011 | 0.011 | [−0.011, 0.033] | −0.003 | 0.009 | [−0.021, 0.015] | 0.014 [−0.014, 0.042] | .325 |
| School mean socioeconomic status | 0.018 | 0.020 | [−0.020, 0.057] | 0.018 | 0.015 | [−0.012, 0.048] | 0.000 [−0.049, 0.049] | 1.000 |
| Proportion female | −0.033 | 0.013 | [−0.058, −0.007] | −0.028 | 0.010 | [−0.049, −0.008] | −0.005 [−0.037, 0.027] | .760 |
| Proportion immigrant background | −0.032 | 0.024 | [−0.078, 0.015] | 0.002 | 0.017 | [−0.031, 0.034] | −0.034 [−0.092, 0.024] | .248 |
| Intercept | 0.206 | 0.033 | [0.141, 0.271] | −0.133 | 0.024 | [−0.181, −0.086] | 0.339 [0.259, 0.419] | < .001 |
| Residual variance | 0.018 | 0.004 | [0.011, 0.025] | 0.018 | 0.003 | [0.012, 0.023] | 0.000 [−0.010, 0.010] | 1.000 |
| *Panel C. Between (school) level — outcome: random slope of attitudes on perpetration* | | | | | | | | |
| Mean slope (intercept of S) | 0.101 | 0.010 | [0.082, 0.120] | 0.056 | 0.008 | [0.040, 0.071] | 0.045 [0.020, 0.070] | < .001 |
| School relational cohesion (cross-level moderation) | 0.018 | 0.011 | [−0.004, 0.039] | −0.005 | 0.008 | [−0.020, 0.009] | 0.023 [−0.004, 0.050] | .091 |
| School institutional order | 0.001 | 0.009 | [−0.017, 0.019] | −0.004 | 0.007 | [−0.017, 0.010] | 0.005 [−0.017, 0.027] | .661 |
| School mean socioeconomic status | 0.013 | 0.011 | [−0.007, 0.034] | −0.005 | 0.008 | [−0.021, 0.011] | 0.018 [−0.009, 0.045] | .186 |
| Residual variance of slope | 0.012 | 0.004 | [0.005, 0.020] | 0.006 | 0.002 | [0.002, 0.011] | 0.006 [−0.003, 0.015] | .180 |
| Covariance of slope with perpetration | 0.006 | 0.003 | [0.000, 0.011] | 0.009 | 0.002 | [0.006, 0.013] | −0.003 [−0.010, 0.004] | .405 |
| *Panel D. Attitude-to-perpetration slope at levels of school relational cohesion* | | | | | | | | |
| −1 SD | 0.083 | 0.015 | [0.053, 0.113] | 0.061 | 0.012 | [0.037, 0.085] | 0.022 [−0.016, 0.060] | .252 |
| Mean | 0.101 | 0.010 | [0.082, 0.120] | 0.056 | 0.008 | [0.040, 0.071] | 0.045 [0.020, 0.070] | < .001 |
| +1 SD | 0.119 | 0.014 | [0.091, 0.146] | 0.050 | 0.009 | [0.032, 0.069] | 0.069 [0.036, 0.102] | < .001 |
| Change from −1 SD to +1 SD | 0.036 | 0.022 | [−0.007, 0.079] | −0.010 | 0.016 | [−0.041, 0.021] | 0.046 [−0.007, 0.099] | .091 |
| *Panel E. Site fixed effects (Bogotá = reference) — outcome: school mean perpetration* | | | | | | | | |
| Bulgaria | 0.261 | 0.071 | [0.121, 0.401] | 0.280 | 0.061 | [0.159, 0.400] | −0.019 [−0.202, 0.164] | .839 |
| Chile | −0.113 | 0.043 | [−0.197, −0.028] | −0.080 | 0.031 | [−0.140, −0.020] | −0.033 [−0.137, 0.071] | .534 |
| Delhi (India) | 0.546 | 0.062 | [0.425, 0.667] | 0.702 | 0.063 | [0.580, 0.825] | −0.156 [−0.329, 0.017] | .078 |
| Dubai (United Arab Emirates) | −0.204 | 0.092 | [−0.384, −0.024] | −0.198 | 0.060 | [−0.316, −0.081] | −0.006 [−0.221, 0.209] | .956 |
| Gunma (Japan) | −0.424 | 0.041 | [−0.504, −0.343] | −0.268 | 0.028 | [−0.324, −0.213] | −0.156 [−0.253, −0.059] | .002 |
| Helsinki (Finland) | −0.222 | 0.063 | [−0.345, −0.099] | −0.190 | 0.043 | [−0.274, −0.106] | −0.032 [−0.182, 0.118] | .675 |
| Jinan (China) | −0.349 | 0.042 | [−0.432, −0.267] | −0.247 | 0.030 | [−0.306, −0.188] | −0.102 [−0.203, −0.001] | .048 |
| Kudus (Indonesia) | −0.032 | 0.064 | [−0.158, 0.094] | −0.090 | 0.042 | [−0.172, −0.008] | 0.058 [−0.092, 0.208] | .449 |
| Peru | −0.201 | 0.043 | [−0.286, −0.117] | −0.131 | 0.035 | [−0.199, −0.063] | −0.070 [−0.179, 0.039] | .207 |
| Sobral (Brazil) | −0.046 | 0.051 | [−0.146, 0.055] | −0.125 | 0.044 | [−0.211, −0.038] | 0.079 [−0.053, 0.211] | .241 |
| Spain | −0.193 | 0.049 | [−0.288, −0.097] | −0.183 | 0.032 | [−0.247, −0.119] | −0.010 [−0.125, 0.105] | .864 |
| Turin (Italy) | −0.237 | 0.054 | [−0.344, −0.131] | −0.159 | 0.044 | [−0.246, −0.073] | −0.078 [−0.215, 0.059] | .263 |
| Emilia-Romagna (Italy) | −0.253 | 0.049 | [−0.348, −0.158] | −0.162 | 0.040 | [−0.241, −0.082] | −0.091 [−0.215, 0.033] | .150 |
| Ukraine (19 of 27 regions) | −0.282 | 0.053 | [−0.386, −0.178] | −0.060 | 0.036 | [−0.129, 0.010] | −0.222 [−0.348, −0.096] | < .001 |
| *Model information* | | | | | | | | |
| Students (n) | 19,063 | | | 19,135 | | | — | |
| Schools (n) | 1,105 | | | 1,104 | | | — | |
| Free parameters | 33 | | | 33 | | | — | |
| Log-likelihood (H0) | −26,987.20 | | | −22,613.04 | | | — | |
| MLR scaling correction factor | 1.193 | | | 1.329 | | | — | |
| Akaike information criterion | 54,040.41 | | | 45,292.08 | | | — | |
| Bayesian information criterion | 54,299.64 | | | 45,551.44 | | | — | |

*Note.* Two-level random-slope models estimated separately in each subsample with maximum likelihood with robust standard errors, schools as clusters. The outcome is the bullying-perpetration index; S denotes the random within-school slope of gender-bias attitudes on perpetration. Student-level covariates are specified as within-level variables; school-level covariates and the 14 site indicators are between-level variables. Differences are Wald tests that treat the two subsamples as independent; because boys and girls attend the same schools, the between-level contrasts are approximate and are reported descriptively rather than as formal significance tests. Panel D reports the attitude-to-perpetration slope evaluated at −1 SD, the mean, and +1 SD of school relational cohesion, computed by MODEL CONSTRAINT. The two subsamples together comprise 38,198 of the 40,880 students in the site-specific analysis (Table A6); the difference reflects listwise deletion on the school-level covariates and on gender.

**Table A5 Frequency of Endorsement of Each Bullying-Perpetration Item, Overall and by Gender (%)**

|  | | **Total sample** | | **Boys** | | **Girls** | | **Boys vs. girls** | |
| --- | --- | --- | --- | --- | --- | --- | --- | --- | --- |
| *Item* | *Wording* | Any | Repeated | Any | Repeated | Any | Repeated | Diff. | Ratio |
| STQM15801 | I left other students out of things on purpose | 20.6 | 6.4 | 24.1 | 7.8 | 16.8 | 5.0 | 7.3 | 1.43 |
| STQM15802 | I made fun of other students | 27.2 | 9.8 | 35.0 | 13.4 | 19.5 | 6.1 | 15.4 | 1.79 |
| STQM15803 | I threatened another student | 11.3 | 5.1 | 15.4 | 6.9 | 7.0 | 3.3 | 8.4 | 2.19 |
| STQM15804 | I took away or destroyed things that belonged to other students | 12.0 | 4.8 | 16.6 | 6.5 | 7.2 | 3.0 | 9.4 | 2.30 |
| STQM15805 | I hit or pushed other students around | 14.4 | 5.8 | 20.1 | 8.0 | 8.6 | 3.5 | 11.5 | 2.34 |
| STQM15806 | I spread nasty rumours about other students | 11.5 | 4.7 | 14.2 | 5.9 | 8.6 | 3.5 | 5.5 | 1.64 |

*Note.* Items are the six ST_BULLYPERP items from question STQM158 of the OECD Survey on Social and Emotional Skills 2023 student questionnaire, reproduced verbatim. The response scale has four options: never or almost never, a few times a year, a few times a month, and once a week or more. “Any” is the percentage endorsing a few times a year or more often; “Repeated” is the percentage endorsing a few times a month or more often. Diff. is the boys’ minus the girls’ “Any” percentage in percentage points; Ratio is the boys’ “Any” percentage divided by the girls’ “Any” percentage.

**Table A6 Site-Specific Within-School Slopes of Gender-Bias Attitudes on Bullying Perpetration, With Random-Effects Meta-Analytic Pooling**

|  | | | **Stata (site-by-site mixed models)** | | | **Mplus (multi-group)** | |  |
| --- | --- | --- | --- | --- | --- | --- | --- | --- |
| *Education system* | Students | Schools | *b* | *SE* | 95% CI | *b* | *SE* | Weight (%) |
| Bogotá (Colombia) | 3,358 | 98 | 0.056 | 0.016 | [0.024, 0.087] | 0.056 | 0.016 | 8.25 |
| Bulgaria | 2,290 | 80 | −0.048 | 0.035 | [−0.117, 0.021] | −0.048 | 0.036 | 4.72 |
| Chile | 3,111 | 95 | 0.082 | 0.015 | [0.052, 0.113] | 0.082 | 0.015 | 8.38 |
| Delhi (India) | 2,350 | 75 | 0.113 | 0.042 | [0.031, 0.195] | 0.113 | 0.039 | 3.86 |
| Dubai (United Arab Emirates) | 4,896 | 152 | 0.110 | 0.017 | [0.077, 0.143] | 0.110 | 0.018 | 8.09 |
| Gunma (Japan) | 3,261 | 78 | 0.035 | 0.012 | [0.011, 0.059] | 0.035 | 0.011 | 8.98 |
| Helsinki (Finland) | 2,000 | 55 | 0.146 | 0.026 | [0.095, 0.197] | 0.146 | 0.026 | 6.28 |
| Jinan (China) | 3,101 | 75 | 0.057 | 0.018 | [0.021, 0.092] | 0.057 | 0.018 | 7.86 |
| Kudus (Indonesia) | 3,043 | 130 | 0.089 | 0.031 | [0.028, 0.151] | 0.089 | 0.031 | 5.30 |
| Peru | 2,003 | 89 | 0.099 | 0.025 | [0.050, 0.149] | 0.099 | 0.026 | 6.41 |
| Sobral (Brazil) | 1,797 | 24 | 0.151 | 0.044 | [0.064, 0.237] | 0.151 | 0.041 | 3.62 |
| Spain | 2,831 | 79 | 0.090 | 0.018 | [0.055, 0.124] | 0.090 | 0.018 | 7.94 |
| Turin (Italy) | 2,648 | 68 | 0.113 | 0.022 | [0.069, 0.156] | 0.113 | 0.024 | 7.03 |
| Emilia-Romagna (Italy) | 2,610 | 66 | 0.071 | 0.023 | [0.027, 0.115] | 0.071 | 0.024 | 6.93 |
| Ukraine (19 of 27 regions) | 1,581 | 89 | 0.063 | 0.025 | [0.013, 0.113] | 0.064 | 0.025 | 6.36 |
| **Random-effects pooled (REML)** | 40,880 | 1,253 | **0.080** | 0.010 | **[0.059, 0.100]** | z = 7.67, p < .001 | | |
| Common-effect pooled | 40,880 | 1,253 | 0.074 | 0.005 | [0.064, 0.085] | Reported for comparison | | |
| **95% prediction interval** |  |  |  |  | **[0.006, 0.153]** | Excludes zero | | |
| Heterogeneity: *τ*² = 0.0011, *τ* = 0.032, *Q*(14) = 46.08, *p* < .001, *I*² = 71.0%, *H*² = 3.45. | | | | | | | | |

*Note.* Each site-specific slope is the mean within-school regression coefficient of bullying perpetration on gender-bias attitudes, with the student-level covariate set of Model 3 and schools as clusters. Two estimation paths are reported: separate two-level random-slope models fitted one system at a time in Stata, and a single 15-group two-level random-slope model fitted in Mplus with maximum likelihood with robust standard errors (180 free parameters, log-likelihood −52,646.54). The two paths agree to within 0.0006 on every system. Pooling uses restricted maximum likelihood on the Stata estimates; weights are inverse-variance weights under the random-effects model. The prediction interval is the Higgins–Thompson interval on k − 2 degrees of freedom and describes the range within which the slope in a new comparable education system would be expected to fall. Fourteen of the 15 confidence intervals lie entirely above zero; the exception is Bulgaria. Mexico, the sixteenth SSES 2023 site, is not included: the OECD reports its data separately from the international average because sampling and technical standards were not met. Ukraine covers 19 of its 27 regions.

**Table A7 Item-Level Invariance of the Gender-Bias Scale and the Feasibility of an Anchored Common Metric Across the 15 Education Systems**

*Panel A. Approximate invariance of each item under free alignment*

| **Item** | **Systems invariant on the intercept** | **Intercept invariance index** | **Systems invariant on the loading** | **Loading invariance index** | **Systems invariant on both** |
| --- | --- | --- | --- | --- | --- |
| *Agentic and status items* | | | | | |
| Ambition | 6 | .000 | 7 | .122 | 4 |
| Leadership | 8 | .855 | 8 | .050 | 4 |
| Technology | 7 | .667 | 7 | .095 | 4 |
| Aggressiveness | 4 | .199 | 13 | .840 | 2 |
| *Communal and care items* | | | | | |
| Caring | 5 | .776 | 12 | .451 | 4 |
| Empathy | 14 | .960 | 4 | .356 | 4 |
| Arts | 7 | .820 | 5 | .363 | 4 |
| Sensitivity | 8 | .844 | 9 | .477 | 6 |
| **Noninvariant, all systems** | **61 of 120 (50.8%)** | | **55 of 120 (45.8%)** | | **116 of 240 (48.3%)** |

*Panel B. Number of noninvariant measurement parameters in each education system*

| **Education system** | **Intercepts (of 8)** | **Loadings (of 8)** | **Total (of 16)** |
| --- | --- | --- | --- |
| Bogotá (Colombia) | 6 | 6 | 12 |
| Peru | 6 | 5 | 11 |
| Chile | 3 | 7 | 10 |
| Gunma (Japan) | 5 | 5 | 10 |
| Sobral (Brazil) | 5 | 5 | 10 |
| Spain | 5 | 4 | 9 |
| Bulgaria | 3 | 5 | 8 |
| Delhi (India) | 5 | 3 | 8 |
| Dubai (United Arab Emirates) | 2 | 6 | 8 |
| Helsinki (Finland) | 5 | 2 | 7 |
| Jinan (China) | 5 | 2 | 7 |
| Kudus (Indonesia) | 5 | 1 | 6 |
| Ukraine (19 of 27 regions) | 4 | 2 | 6 |
| Turin (Italy) | 1 | 1 | 2 |
| Emilia-Romagna (Italy) | 1 | 1 | 2 |

*Panel C. Fit of the fully invariant one-factor multiple-group model*

| **Fit statistic** | **Value** |
| --- | --- |
| Free parameters | 164 |
| Chi-square (df = 496) | 33,831.29 |
| Scaling correction factor for MLR | 1.5064 |
| Comparative fit index | .699 |
| Tucker-Lewis index | .745 |
| Root mean square error of approximation | .146 [.145, .148] |
| Standardized root mean square residual | .131 |
| Loglikelihood, H0 and H1 | −504,467.65 and −478,986.11 |
| Students, systems, schools | 47,165, 15, 1,262 |

*Note.* Panel A summarizes the free alignment solution reported in Table 2, estimated with maximum likelihood with robust standard errors across 15 known classes, N = 47,165. Item labels are those used in Table 2. Systems invariant is the number of the 15 education systems in which Mplus judged the parameter approximately invariant. The invariance index is the R-squared printed by the alignment procedure for that parameter. It is computed over the systems in which the parameter held approximate invariance, and it gives the share of the remaining variation in that parameter that is accounted for by differences in the factor mean and the factor variance. A value near 1 means that a system’s value on the parameter can be predicted from where that system sits on the latent trait. A value of .000 for the ambition intercept is a printed result and not a missing entry, and it means that none of the residual variation in that intercept is accounted for in this way. Systems invariant on both counts the systems in which an item held its loading and its intercept at the same time, which is what an anchor item has to do. The conventional requirement is at least 13 of the 15 systems, and no item met it. Panel B reports how many of each system’s 16 measurement parameters were flagged, counting 8 intercepts and 8 loadings. Panel C reports a one-factor multiple-group confirmatory factor analysis in which every loading and every intercept is constrained equal across the 15 systems and the factor mean is free in 14 of them, estimated with maximum likelihood with robust standard errors and schools as clusters. Two cautions apply to Panel A. Flags rest on significance tests and are sensitive at this sample size, and the alignment run does not adjust for the nesting of students within schools, so the flags lean toward finding noninvariance. Mexico, the sixteenth system in the Survey on Social and Emotional Skills 2023, is not analyzed.

**Table A8 Site-Specific Within-School Slopes Estimated on the OECD Index and on the Harmonized (Aligned) Metric**

|  | **OECD index** | | **Harmonized metric** | |  |
| --- | --- | --- | --- | --- | --- |
| **Education system** | **b (SE)** | **95% CI** | **b (SE)** | **95% CI** | **Change** |
| Bogotá (Colombia) | .056 (.017) | [.023, .089] | .059 (.016) | [.028, .091] | +.003 |
| Bulgaria | −.046 (.035) | [−.116, .023] | −.093 (.035) | [−.162, −.025] | −.047 |
| Chile | .085 (.016) | [.054, .116] | .090 (.016) | [.058, .122] | +.005 |
| Delhi (India) | .113 (.042) | [.030, .196] | .164 (.037) | [.091, .237] | +.051 |
| Dubai (United Arab Emirates) | .108 (.017) | [.074, .142] | .153 (.020) | [.114, .192] | +.044 |
| Gunma (Japan) | .033 (.012) | [.009, .058] | .030 (.012) | [.006, .055] | −.003 |
| Helsinki (Finland) | .146 (.027) | [.092, .199] | .141 (.028) | [.086, .195] | −.005 |
| Jinan (China) | .054 (.018) | [.019, .089] | .061 (.017) | [.027, .095] | +.007 |
| Kudus (Indonesia) | .083 (.032) | [.021, .145] | .088 (.036) | [.017, .158] | +.004 |
| Peru | .106 (.027) | [.054, .158] | .097 (.025) | [.049, .145] | −.009 |
| Sobral (Brazil) | .146 (.044) | [.060, .232] | .099 (.036) | [.029, .170] | −.047 |
| Spain | .082 (.017) | [.048, .116] | .097 (.017) | [.063, .131] | +.015 |
| Turin (Italy) | .100 (.023) | [.054, .146] | .111 (.025) | [.062, .160] | +.011 |
| Emilia-Romagna (Italy) | .061 (.023) | [.015, .107] | .091 (.024) | [.043, .138] | +.030 |
| Ukraine (19 of 27 regions) | .061 (.027) | [.008, .114] | .065 (.029) | [.008, .121] | +.004 |
| **Random-effects pooled (REML)** | **.077 (.010), [.057, .097]** | | **.083 (.014), [.056, .111]** | |  |
| Common-effect pooled | .071 (.005), [.061, .082] | | .077 (.005), [.067, .088] | |  |
| Test of the pooled slope | z = 7.52, p < .001 | | z = 5.92, p < .001 | |  |
| 95% prediction interval | [.006, .148] | | [−.026, .193] | |  |
| Between-site variance (τ²) | .0010 | | .0024 | |  |
| I² | 68.4% | | 83.7% | |  |
| H² | 3.16 | | 6.13 | |  |
| Test of homogeneity | Q(14) = 42.77, p < .001 | | Q(14) = 70.45, p < .001 | |  |
| Students | 40,880 | | 40,899 | |  |
| Schools | 1,253 | | 1,253 | |  |

*Note.* Each slope is the mean within-school regression coefficient of bullying perpetration on gender-bias attitudes, estimated one education system at a time with the student-level covariate set of Model 3, a random slope, and schools as clusters. Both columns come from a single run under one specification, so the comparison across columns does not depend on estimation settings. The harmonized metric is the factor score from the free alignment solution reported in Table 2 and Appendix Table A7, standardized once in the pooled sample. The two versions of the attitude measure correlate .947 at the student level. Across the 15 systems the two sets of slope estimates correlate .90, and their rank correlation is .89. Change is the harmonized estimate minus the OECD index estimate. Student and school counts in the OECD index column match Appendix Table A6 in every system, and the estimates in that column differ from Appendix Table A6 by no more than .013. The harmonized column covers 19 more students, because the aligned factor score is defined for any student who answered at least one of the eight items. Pooling uses restricted maximum likelihood, and weights are inverse-variance weights under the random-effects model. The prediction interval is the Higgins-Thompson interval on k − 2 degrees of freedom. Fourteen of the 15 confidence intervals lie entirely above zero on each metric. Bulgaria is the exception on both, and its slope is reliably negative on the harmonized metric. Mexico, the sixteenth system, is not analyzed.

**Supplementary Figures**

**Figure S1 Site-Specific Within-School Slopes of Gender-Bias Attitudes on Bullying Perpetration on the OECD Reporting Metric**

*Note.* Random-effects meta-analysis by restricted maximum likelihood across 15 education systems. Squares are the site-specific slopes with square size proportional to study weight, horizontal lines are 95% confidence intervals, the diamond is the pooled estimate, and the red line is the 95% prediction interval. Estimates and heterogeneity statistics are reported in Appendix Table A6.

**Figure S2 Site-Specific Within-School Slopes of Gender-Bias Attitudes on Bullying Perpetration on the Harmonized (Aligned) Metric**

*Note.* Random-effects meta-analysis by restricted maximum likelihood across 15 education systems, with the gender-bias scale placed on a common metric by alignment. Estimates and heterogeneity statistics are reported in Appendix Table A8. The pooled association is close to the value obtained on the OECD index, while the variation between systems is larger and the 95% prediction interval includes zero.

**Figure S3 Gender-Differentiated Slope of Gender-Bias Attitudes on Bullying Perpetration at Low and High School Relational Cohesion (Model 4)**

*Note.* Points are the estimated within-school slope of gender-bias attitudes on bullying perpetration for boys (solid line) and girls (dashed line) at low (−1 standard deviation) and high (+1 standard deviation) school relational cohesion, and bars are 95% confidence intervals. The three-way interaction (relational cohesion × attitudes × female) reached the conventional threshold (b = −.025, SE = .012, p = .046) but is treated as exploratory throughout, since split-sample models did not reproduce it as a reliable effect (Appendix Table A4).
